# Rabies Virus Glycoprotein G Variation Is Associated with Neurovirulence and Reduced Vamp2 Abundance in a Mouse Model of Direct CNS Infection

**DOI:** 10.3390/vetsci13090919

**Published:** 2026-09-07

**Authors:** Chunyu Liu, Hao Guo, Kun Yin, Yuming Sun, Zipeng Ma, Fengxue Wang, Yan Niu, Yongjun Wen

**Affiliations:** 1 College of Basic Medicine, Inner Mongolia Medical University, Hohhot 010110, China; chunyuliu_vet@163.com; 2 College of Veterinary Medicine, Inner Mongolia Agricultural University, Hohhot 010018, China; wangfx_vet@163.com; 3 College of Life Sciences, Inner Mongolia University, Hohhot 010020, China; gh19960627@126.com; 4 Zhoukou Academy of Agricultural Sciences, Zhoukou 466001, China; yinkun18@126.com; 5 Yulin Animal Husbandry and Veterinary Service Center, Yulin 719000, China; sym911105@sina.com; 6 Yulin Animal Husbandry Comprehensive Service Station, Yulin 719000, China; mzp201955@163.com

**Keywords:** rabies, G protein, primary mouse neurons, synaptic vesicle, Vamp2

## Abstract

Rabies virus causes fatal neurological disease, but the mechanisms by which its glycoprotein G influences neuronal dysfunction remain incompletely understood. In this study, we rescued two recombinant RABV LBNSE strains carrying G proteins from either the attenuated SAD-B19 strain or the virulent CVS11 strain, designated as rLBNSE-SfG and rLBNSE-CfG, respectively. Infection of primary mouse neurons and challenge experiments in mice showed that rLBNSE-CfG was associated with increased early neuronal infectivity and greater neurovirulence after intracerebral (i.c.) injection. Western blot analysis revealed that the SAD-B19-derived G protein exhibited a higher apparent molecular weight than the CVS11-derived G protein. Bioinformatic analysis identified strain-specific differences in putative post-translational modification sites, which may contribute to the observed migration difference. Quantitative proteomic analysis of mouse brain tissue showed that proteins downregulated in the rLBNSE-CfG group were enriched in synaptic vesicle cycle-related pathways and synapse-associated Gene Ontology (GO) terms. Integrated analysis identified a SNARE-associated module containing Snap25, Stx1a, and Vamp2, among which Vamp2 was significantly reduced in the rLBNSE-CfG group. In primary neurons, reduced Vamp2 abundance was associated with the extracellular domain of CVS11 G protein. These findings associate CVS11-derived G protein variation with reduced Vamp2 abundance and a presynaptic vesicle-related proteomic signature, although whether these molecular alterations directly impair synaptic vesicle release remains to be determined.

## Simple Summary

Rabies virus infection causes a severe and usually fatal neurological disease, but the viral determinants underlying differences in neurovirulence remain incompletely understood. The rabies virus glycoprotein G is exposed on the viral surface and plays essential roles in viral entry, spread, and host–cell interactions. In this study, recombinant RABVs carrying G proteins from the attenuated SAD-B19 strain or the more virulent CVS11 strain were rescued within the same LBNSE genetic background. Although G replacement did not significantly affect viral replication in cultured cells, rLBNSE-CfG exhibited enhanced neurovirulence after intracerebral injection and increased early infection of primary neurons. Proteomic analysis of mouse brain tissue revealed that rLBNSE-CfG was associated with reduced expression of proteins involved in the synaptic vesicle cycle and neurotransmitter release, including a SNARE-associated module containing Snap25, Stx1a, and Vamp2. In primary neurons, decreased Vamp2 abundance was associated with rLBNSE-CfG infection and expression of the CVS11-G extracellular-domain construct. Immunofluorescence confirmed the neuronal expression and distribution of CVS11-G domain constructs in Map2-positive neurons. These findings associate CVS11-derived G variation with reduced Vamp2 abundance and a presynaptic vesicle-related proteomic signature, although whether these molecular alterations directly affect synaptic vesicle release remains to be determined.

## 1. Introduction

Rabies is an ancient, lethal zoonotic disease that causes severe and usually fatal encephalomyelitis. With a case-fatality rate approaching 100%, it poses a serious threat to global public health [1,2]. The disease is primarily transmitted by bites from animals infected with rabies virus (RABV), including dogs, cats, foxes, and bats. Rabies is estimated to cause approximately 59,000 human deaths annually in more than 150 countries, with the highest burden in Africa and Asia [3,4]. RABV is a single-stranded, negative-sense, nonsegmented RNA virus measuring approximately 100–300 nm long and about 75 nm wide. Under electron microscopy, mature virions are bullet-shaped with a bluntly rounded end and a concave end [5], and their outer shell is a bilayered lipid membrane [6]. The RABV genome is about 12 kb and encodes, sequentially from the 3′ to 5′ end, nucleocapsid protein (N), phosphoprotein (P), matrix protein (M), glycoprotein (G), and RNA-dependent RNA polymerase (L) [7,8]. The G protein is the only structural protein on the viral envelope that elicits neutralizing antibodies, and it is the key determinant of viral neurotropism, synaptic transmission capacity, and pathogenicity [9,10,11]. However, the specific molecular mechanisms by which G proteins from different RABV strains regulate viral replication dynamics in neurons, alter synaptic function, and modulate the expression of related host proteins remain incompletely understood.

RABV pathogenicity, neurotropism, and resultant neurological damage depend on interactions between the virus and the host nervous system, notably receptor-mediated entry, retrograde axonal transport, synaptic transmission, and intracellular replication. The G protein, the sole glycosylated protein exposed on the virion surface, mediates receptor binding, membrane fusion, and viral spread along neural circuits. Moreover, G proteins from different RABV strains have different glycosylation sites [12]. Prior work has shown an inverse relationship between G expression and RABV neurovirulence after direct CNS exposure: pathogenic strains limit G expression or its incorporation into mature virions to evade immune recognition, whereas attenuated strains often show higher G expression and stronger immune activation [13,14]. Consistently, mutations that increase G expression in SAD L16 or that alter key residues in G have led to reduced viral pathogenicity [15,16,17]. Structural features of the G protein, including strain-specific N-glycosylation sites and amino acid substitutions, also affect viral replication, immune activation, internalization, and virulence. For example, N-glycosylation at particular asparagine residues has been linked to enhanced viral proliferation and reduced pathogenicity, whereas the Gly_349_ to Glu_349_ substitution has attenuated RABV by enhancing immune responses [18,19,20]. Residues in the G protein cytoplasmic tail, including S_469_, F_474_, G_475_, and Y_497_, regulate viral internalization [21]. Beyond its direct roles in viral entry, replication, and immune evasion, the G protein also contributes to neuronal tropism and synaptic spread. RABV strains pseudotyped or enveloped with different G proteins have displayed distinct retrograde synaptic transfer, neuronal tropism, and circuit-tracing efficiency, indicating that structural differences in G protein can reshape virus–neuron interactions [22,23,24,25]. Most studies of RABV neurovirulence after direct CNS exposure focus on viral replication and immune modulation, while whether G proteins from virulent and attenuated strains differentially regulate host neuronal proteins involved in synaptic vesicle recycling, membrane fusion, and neurotransmitter release has received less attention. Because Vamp2 is a core component of the synaptic vesicle SNARE machinery, changes in Vamp2 abundance and related synaptic proteins may serve as potential molecular correlates of altered presynaptic vesicle-related processes associated with different RABV G protein-mediated neurovirulence phenotypes. However, whether these molecular alterations directly contribute to functional synaptic impairment remains to be determined.

RABV CVS11 is a highly virulent standard strain derived from PV, used for challenge modeling [21], and RABV SAD-B19 is a highly attenuated vaccine strain derived from SDA, used to produce oral rabies vaccines that are widely administered worldwide [26,27,28]. In the last century, SAD-B19 was successfully used for oral immunization of foxes in some European countries. However, reports have shown that this strain is pathogenic in rodents [29,30]. The Arg333 Glu mutation in the SAD-B19 G protein greatly reduced its pathogenicity, and this has been used as a wildlife vaccine strain in many European countries [31]. Furthermore, studies have shown that immunized dogs produce only low levels of neutralizing antibodies [32]. The RABV LBNSE strain was generated by removing the pseudogene from the SAD-L16 strain and introducing BsiWI and NheI restriction sites between the G and L genes [33], together with the Asn194 to Ser194 and Arg333 to Glu333 mutations [34].

To assess how G proteins from different RABV strains influence neurovirulence within the same genetic background, we generated two recombinants by replacing the G gene in the LBNSE backbone with the G gene from either the attenuated SAD-B19 strain or the virulent CVS11 strain. The native signal peptide of each G protein was preserved, and a Flag tag was inserted immediately downstream of the predicted signal peptide cleavage site at the N terminus of the mature G ectodomain. The rescued recombinants were named rLBNSE-SfG and rLBNSE-CfG, respectively, and were used to systematically compare viral growth, replication, neurotropism, and viral protein expression. G protein replacement did not significantly alter viral titers, and no clear differences in G or N protein expression were observed between rLBNSE-SfG and rLBNSE-CfG in primary neurons, indicating that the distinct neurovirulent phenotypes were not simply due to altered viral replication or viral protein abundance. By contrast, rLBNSE-CfG produced stronger neurovirulence effects than rLBNSE-SfG. Because the in vivo comparison was performed using intracerebral (i.c.) injection, the mouse experiment was designed to evaluate neurovirulence after direct CNS exposure rather than the complete process of natural peripheral rabies infection. Proteomic analysis revealed substantial remodeling of host neuronal proteins. GO enrichment analysis showed that proteins downregulated in the rLBNSE-CfG group were primarily associated with the synaptic vesicle cycle, neurotransmitter secretion, calcium-ion-regulated exocytosis, presynapse, synaptic vesicle membrane, SNARE binding, and syntaxin binding. KEGG pathway analysis identified the synaptic vesicle cycle as a key enriched pathway, along with synapse- and neurodegeneration-related pathways, suggesting that the CVS11 G protein is associated with proteomic downregulation of presynaptic vesicle-cycle-related proteins. Based on these proteomic findings, we focused on the core SNARE-associated proteins Vamp2, Stx1a, and Snap25, which mediate synaptic vesicle fusion with the presynaptic membrane. Among them, Vamp2 was significantly downregulated in the rLBNSE-CfG group compared with the rLBNSE-SfG group, and domain analysis indicated that this effect was mainly associated with the extracellular domain of the CVS11 G protein. These results identify a Vamp2-associated presynaptic proteomic signature linked to rLBNSE-CfG challenge, suggesting a potential molecular association with altered presynaptic vesicle-related processes, although the functional consequences for synaptic vesicle release remain to be determined.

## 2. Materials and Methods

### 2.1. Plasmids, Viruses, and Cells

The RABV full-length plasmid pCDNA3.1-LBNSE and the infectious clonal helper plasmids pCDNA3.1-N, pCDNA3.1-P, pCDNA3.1-G, and pCDNA3.1-L were obtained from previous laboratory studies [33]. The weakly virulent RABV strain SAD-B19 and the laboratory-fixed strain CVS11 were gifted by Prof. Zhenfang Fu. BSR cells, a cloned line derived from BHK-21 (Baby Hamster Syrian Kidney) cells, were cultured in Dulbecco’s modified Eagle’s medium (DMEM, Gibco, Grand Island, NY, USA). Mouse neuroblastoma (mNA) cells were cultured in RPMI 1640 medium (Gibco, Grand Island, NY, USA). All cell lines were purchased from the National Biomedical Laboratory Cell Repository (BMCR, Beijing, China). Media were supplemented with 10% heat-inactivated fetal bovine serum (FBS, Gibco, Grand Island, NY, USA) and 1% penicillin-streptomycin (PS, Gibco, Grand Island, NY, USA).

### 2.2. Construction of Plasmid r-pLBNSE

We amplified the G gene from RABV strains of differing virulence using RABV-G F/R primers. Through overlap PCR, the *EcoR*I sites at nucleotide positions 1126 and 1129 were mutated, and the nucleotides at positions 3324 and 3329 were converted to create a *Pst*I site. The *Pst*I sites at nucleotide positions 4961, 4962, and 4965 were also mutated. Based on the full-length pCDNA3.1-LBNSE plasmid sequence, we designed three primer pairs-Fragment-1-F/R (*EcoR*I site underlined), Fragment-2-F/R, and Fragment-3-F/R (*Nhe*I site underlined) (Table 1). The PCR conditions were as follows: initial denaturation at 94 °C for 2 min, then 35 cycles of 98 °C for 10 s, 52 °C for 30 s, and 68 °C for the fragment-specific extension times (Fragment-1-F/R: 1 min 20 s; Fragment-2-F/R: 2 min 20 s; Fragment 3-F/R: 1 min 50 s; and Fragment-1F/3R: 5 min 10 s), with a final extension at 68 °C for 7 min. The plasmid r-pLBNSE was constructed by cloning the PCR product into pCDNA3.1-LBNSE using *EcoR*I (Thermo Fisher Scientific, Waltham, MA, USA; FD0274) and NheI (Thermo Fisher Scientific, Waltham, MA, USA; FD0974). All plasmids were sequenced and verified (BGI Genomics, Beijing, China).

### 2.3. Construction of r-pLBNSE-SfG and r-pLBNSE-CfG cDNAs

**Figure 1:**
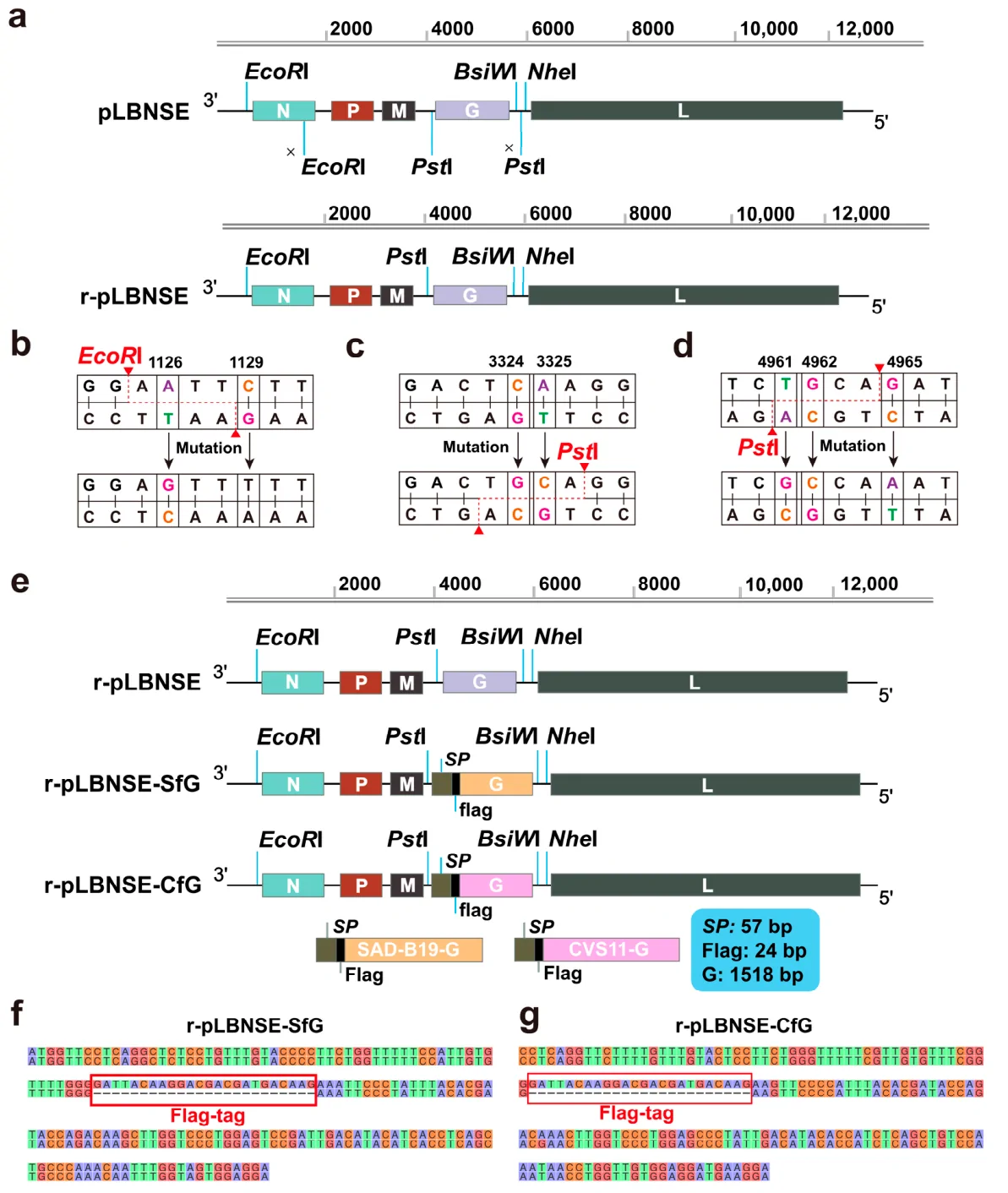
Construction and validation of r-pLBNSE-SfG, r-pLBNSE-CfG, and r-pLBNSE. (**a**) Schematic of r-pLBNSE constructs indicating mutations at *EcoR*I and *Pst*I restriction sites; (**b**) mutation of the *EcoR*I site at nucleotide positions 1126 and 1129, with amplification using Fragment1-F/3-R primers and sequencing; (**c**) mutation of the *Pst*I site at nucleotide positions 3324 and 3325; (**d**) mutation of the *Pst*I site at nucleotide positions 4961, 4962, and 4965; (**e**) schematic of G protein substitutions between SAD-B19 and CVS11 strains. Orange: SAD-B19 G protein; pink: CVS11 G protein. In particular, the signal peptide is that of the RABV G protein itself; (**f**,**g**) sequencing results for r-pLBNSE-SfG and r-pLBNSE-CfG, using RV-G-F/R primers and sequencing.

Based on the NCBI-published G protein gene sequences for the weakly virulent strain SAD-B19 (GenBank: M31046.1) and the fixed strain CVS11 (GenBank: GQ918139.1), we designed primers rLBNSE-SfG-F/R and rLBNSE-CfG-F/R (Table 1). The PCR conditions were as follows: 94 °C for 2 min, then 35 cycles of 98 °C for 10 s, 52 °C for 30 s, and 68 °C for 1 min 50 s, followed by a final extension at 68 °C for 7 min. The G protein native signal peptide was fully preserved. The Flag tag sequence (DYKDDDDK) was inserted immediately downstream of the predicted signal peptide cleavage site and upstream of the mature G ectodomain, and *Pst*I and *BsiW*I restriction sites were added at the 5′ end. The tag was positioned at the N terminus of the mature G protein, rather than at the C terminus. The overall organization of the engineered G protein consisted of a signal peptide, a Flag tag, a mature ectodomain, a transmembrane domain, and a cytoplasmic tail. The predicted extracellular domain, transmembrane domain, and cytoplasmic tail were annotated according to the corresponding SAD-B19 and CVS11 G-protein sequences. The amino acid sequences surrounding the signal peptide/Flag junction are presented in Figure 1. The G protein genes from SAD-B19 and CVS11 were amplified separately and cloned into r-pLBNSE using *Pst*I and *BsiW*I to generate the plasmids r-pLBNSE-SfG and r-pLBNSE-CfG. In addition, we synthesized the CVS11 G protein extracellular region (amino acids 28–458) and intracellular region (amino acids 482–524), each with the G protein signal peptide appended at the 5′ end. These synthesized sequences were cloned into pCDNA3.1 using *Hind*III and *Kpn*I and designated as pcDNA3.1-CVS11-G-outside and pcDNA3.1-CVS11-G-inside. For the domain-fragment experiment, primary mouse neurons are transfected with equal amounts of the Flag-only control plasmid, pcDNA3.1-CVS11-G-outside, or pcDNA3.1-CVS11-G-inside using Lipofectamine 3000. The groups are designated Control, Out, and In, respectively. Protein and RNA samples are collected 72 h after transfection. Each group comprises three independent biological replicates.

### 2.4. Rescue and Validation of Recombinants

r-pLBNSE-SfG, r-pLBNSE-CfG, and r-pLBNSE were co-transfected into BSR cells using Lipofectamine^TM^ 3000 Reagent (Thermo Fisher Scientific, Waltham, MA, USA), together with the helper plasmids pCDNA3.1-N, pCDNA3.1-P, pCDNA3.1-G, and pCDNA3.1-L. Each well received 2.0 µg of either r-pLBNSE-SfG, r-pLBNSE-CfG, or r-pLBNSE, plus 0.5 µg of pCDNA3.1-N, 0.25 µg of pCDNA3.1-P, 0.15 µg of pCDNA3.1-G, and 0.1 µg of pCDNA3.1-L. After 6 h, the medium was replaced with fresh DMEM containing 1% FBS and 1% PS, and cells were incubated at 37 °C with 5% CO_2_ for 6 d. The cell suspension was centrifuged at 1500× *g* for 10 min, the supernatant was collected to infect mNA cells, and infected cells were fixed in 80% acetone for 36 h. The recombinants rLBNSE-SfG, rLBNSE-CfG, and rLBNSE were verified with FITC-labeled monoclonal antibodies against the RABV N protein (Fujirebio Diagnostics, Malvern, PA, USA).

### 2.5. Determination of Viral Titers

For viral titer analysis, BSR cells were prepared at 5 × 10^5^ cells/mL and incubated at 37 °C in an atmosphere containing 5% CO_2_. Next, 90 µL of DMEM containing 10% FBS and 1% PS was added to each well of a 96-well plate, and 10 µL of the viral suspension was added to row 1 and serially diluted 10-fold through row 8 and row 9, served as a negative control. Fifty microliters of the BSR cell suspension was added to each well, mixed thoroughly, and cultured at 37 °C in an atmosphere containing 5% CO_2_. After 36 h, cells were fixed in 80% acetone for 25 min. Cells were incubated with FITC-labeled monoclonal antibodies against the RABV N protein (1:50) for 45 min and washed with PBST, and fluorescence focus units (FFUs) were recorded under a fluorescence microscope. The viral titer was calculated using the following formula: FFU = X_0_ − d/2 + d × (n_0_ + n_1_ + … + n_k_)/N. X_0_ = log10 of the dilution factor at the highest dilution showing 100% infected wells (i.e., all wells positive); d = log10 of the serial dilution step (d = 1 for 10-fold serial dilutions); and n_0_, n_1_, ⋯, n_k_ = the number of positive (infected) wells at each dilution, from the highest concentration (n_0_) to the lowest concentration showing any positive wells (n).

### 2.6. Growth Kinetics of Recombinants

BSR and mNA cells were infected with six generation recombinants, including rLBNSE-SfG, rLBNSE-CfG, and rLBNSE, at an MOI of 0.1. Viral samples were collected at 12, 24, 48, 72, and 96 h post-infection, and viral titers were determined at each time point. Multistep growth curves were plotted, and data are presented as the means of three biological replicates.

### 2.7. RABV RNA Extraction

Total RNA was extracted with TRIzol reagent, and BSR and mNA cells were infected with RABV at a multiplicity of infection (MOI) of 0.1. After 72 h, 500 μL of the viral supernatant was collected, mixed with 500 μL of TRIzol, and incubated at room temperature for 10 min. Then, 200 μL of chloroform was added, and the mixture was centrifuged at 13,000 rpm and 4 °C for 15 min. The aqueous phase was collected, and the extraction was repeated by adding an additional 200 μL of chloroform, mixing, and centrifuging again at 13,000 rpm and 4 °C for 15 min. RNA was precipitated by adding an equal volume of isopropanol, performing gentle mixing by inversion, and performing incubation at 4 °C for 30 min. After centrifugation at 12,000 rpm and 4 °C for 10 min, the supernatant was discarded. The RNA pellet was washed with 1 mL of pre-cooled 75% ethanol, followed by centrifugation at 8000 rpm and 4 °C for 10 min. The supernatant was discarded, and the RNA pellet was air-dried for 5–10 min before being dissolved in 20 μL of DEPC-treated water. RNA was reverse-transcribed into cDNA using a reverse transcription kit (Thermo Fisher Scientific, Waltham, MA, USA, K1691). Primary mouse neurons were assigned to four groups: an untreated Control group, a Flag-vector group transfected with the corresponding Flag-tagged control plasmid, an Outside group transfected with pcDNA3.1-CVS11-G-outside, and an inside group transfected with pcDNA3.1-CVS11-G-inside. Total RNA was collected 72 h after transfection. RNA extraction and cDNA synthesis were performed as described above.

### 2.8. RT-qPCR

The copy numbers of the positive standards were calculated from the recombinant plasmid concentration using the copy number formula. The GoTaq qPCR Master Mix kit (Thermo Fisher Scientific, Waltham, MA, USA, A6002) was used, and the reaction system contained 10 μL of SYBR Green I, 0.4 μL of each RV-N-F/R primer, 1 μL of the DNA template, and ddH_2_O, amounting to a final volume of 20 μL. The reaction conditions were as follows: initial denaturation at 95 °C for 30 s, denaturation at 95 °C for 15 s, and annealing/extension at 55 °C for 60 s. A standard curve was generated over 40 cycles, and the copy number of the N gene in each sample was calculated from the CT value of the standard curve. RT-qPCR analysis of *Vamp2* expression was performed using the same reaction system and amplification conditions described above, except that annealing and extension were conducted at 60 °C for 30 s. Gapdh was used as the reference gene, and relative *Vamp2* mRNA expression was calculated using the 2^−ΔΔCt^ method. All data are presented as averages of three biological replicates.

### 2.9. Analysis of Neurovirulence After Direct Intracerebral (i.c.) Injection in Mice

Forty 4-week-old female BALB/c mice (Inner Mongolia Medical University) were randomly assigned to four groups—rLBNSE-SfG, rLBNSE-CfG, rLBNSE, and DMEM—with 10 mice per group. The F6-generation rLBNSE-SfG, rLBNSE-CfG, and rLBNSE were each diluted to 1 × 10^5^ FFU/30 μL in DMEM. Mice were challenged by intracerebral (i.c.) injection with a 30 μL dose, and the DMEM group received 30 μL. Clinical signs and mortality were recorded from 0 to 15 d after challenge. The sample size (10 animals per group) was determined based on the expected large effect size for comparisons of RABV virulence in the brain, consistent with established study designs for RABV pathogenicity [35]. This direct intracerebral (i.c.) injection model was used to compare neurovirulence after central nervous system exposure and was not intended to model peripheral entry or natural rabies transmission.

### 2.10. Isolation and Culture of Mouse Primary Neurons

Embryonic day 16–18 fetuses were obtained from pregnant BALB/c mice purchased from the Inner Mongolia Medical University. After euthanasia, fetal mouse brain tissue was collected, and the cerebral cortex with meninges and vascular membranes removed was transferred to a Petri dish. The tissue was digested 3 times with 0.25% trypsin at 37 °C for 20 min with intermittent shaking. Five milliliters of neurobasal medium containing 10% FBS and 1% PS was added, followed by gentle pipetting 10 times. The resulting neuronal suspension was placed on ice for 2 min, and the supernatant was transferred to a pre-cooled EP tube. After centrifugation at 4 °C and 800 rpm for 5 min, the supernatant was discarded and the pellet was resuspended in a neurobasal medium containing 10% FBS and 1% PS. Neurons were cultured in 6-well plates at a density of 5 × 10^5^ cells/mL. After 4 h, the medium was replaced with neurobasal medium (Gibco, Grand Island, NY, USA) supplemented with 2% B27 (Gibco, Grand Island, NY, USA), 1% glutamine (Gibco, Grand Island, NY, USA), and 1% PS. Morphology was observed after 1, 3, and 7 days of culture.

### 2.11. Validation of Mouse Primary Neurons

Cells were fixed with paraformaldehyde at 4 °C for 1 h and then washed three times with PBS; then, the cells were permeabilized with 3% Triton^TM^ X-100 for 15 min and washed 3 times with PBS. After blocking with 3% bovine serum albumin (BSA) at 37 °C for 1 h, the cells were incubated with anti-Map2 monoclonal antibodies (Abcam, Cambridge, UK; ab32454) for 6 h. The cells were then washed 3 times with PBST and incubated with Cy3-labeled goat anti-rabbit secondary antibodies (Abcam, Cambridge, UK; ab6939) at room temperature for 1 h. After 3 additional washes with PBST, the cells were incubated with DAPI (Abcam, Cambridge, UK; ab104139) for 30 min. Primary neuron purity was assessed by Map2 labeling (ZEISS, HAL100, Oberkochen, Germany).

### 2.12. Detection of CVS11-G Domain Fragment Expression

Primary neurons were transfected with Flag-tagged CVS11-G extracellular-domain construct (Outside), Flag-tagged CVS11-G intracellular-domain construct (Inside), or Flag-vector control (Flag). Forty-eight hours after transfection, cells were fixed with 4% paraformaldehyde and permeabilized with 0.1% Triton X-100. After blocking, cells were incubated overnight at 4 °C with mouse anti-Flag antibody (Abcam, Cambridge, UK; AB18230) and rabbit anti-Map2 antibody (Abcam, Cambridge, UK; ab32454) to detect the expression of CVS11-G domain fragments and neuronal structures, respectively. After washing, cells were incubated with FITC-labeled goat anti-mouse secondary antibody (Abcam, Cambridge, UK; ab6785) and Cy3-labeled goat anti-rabbit secondary antibody (Abcam, Cambridge, UK; ab6939) for 1 h at room temperature. Nuclei were counterstained with DAPI. Fluorescence images were acquired using a fluorescence microscope, and the distribution of Flag-positive signals within Map2-positive neurons was analyzed to evaluate the expression and neuronal distribution of the isolated CVS11-G domain fragments.

### 2.13. Cell Viability Assay

Cell viability was assessed using the Cell Counting Kit-8 (CCK-8) assay. Briefly, cells in the logarithmic growth phase were seeded into culture plates at a density of 1 × 10^4^ cells/mL. After transfection with the indicated constructs, cells were washed with PBS, and 10 μL of 10% diluted CCK-8 solution was added to each well. Cells were incubated for approximately 90 min at 37 °C in a humidified incubator. The absorbance at 450 nm was measured using a microplate reader (Molecular Devices, Sunnyvale, CA, USA). Cell viability was calculated according to the following formula: Cell viability (%) = (OD of treated group/OD of control group) × 100%. The experiment was independently repeated three times.

### 2.14. RABV Infection Rates in Primary Mouse Neurons

Mouse primary neurons were infected with rLBNSE-SfG and rLBNSE-CfG at an MOI of 0.01 and fixed for 20 min at room temperature by adding 80% cold acetone at 24, 48, 72, and 96 h. Neurons were incubated with an anti-intracerebral (i.c.) monoclonal antibody (1:100) overnight at 4 °C and washed three times with PBST. The Cy3-labeled goat anti-rabbit secondary antibody was added, and samples were incubated for 1 h at room temperature. After washing with PBST, the FITC-labeled RABV N protein antibody (1:50) was applied and samples were incubated at room temperature for 1 h. RABV-infected primary neurons were then detected by immunofluorescence after DAPI staining. Each group consisted of three biological replicates. In each well, three fields of view were randomly selected for counting, and the percentage of RABV N-protein-positive neurons relative to Map2-positive neurons was calculated. The entire infection experiment was repeated independently three times.

### 2.15. Western Blotting

Recombinants that had been correctly identified were inoculated into BSR cells or mouse primary neurons, and total proteins were extracted separately after 48 h using a RIPA lysis buffer (Beyotime, Shanghai, China). Protein concentrations were determined using a Pierce^TM^ BCA Protein Assay Kit (Thermo Fisher Scientific, Waltham, MA, USA; TK274303). Proteins were denatured, separated by SDS-PAGE, and transferred to polyvinylidene difluoride membranes. Membranes were blocked with 5% skim milk for 2 h and washed 3 times with TBST. Membranes were then incubated with a rabbit anti-Flag polyclonal antibody (Abcam, Cambridge, UK; ab1162) for 10 h at 4 °C, washed 3 times with TBST, incubated with goat anti-rabbit IgG H&L (HRP) (Abcam, Cambridge, UK; ab6721) for 1 h, and washed 3 times with TBST. G protein expression was analyzed using an enhanced chemiluminescence chromogenic solution (Thermo Fisher Scientific, Waltham, MA, USA; SK255593) and a protein-scanning imager.

### 2.16. Collection of Mouse Brain Tissue

Nine 4-week-old female BALB/c mice (Inner Mongolia Medical University) were divided into three groups of three. Mice received rLBNSE-SfG, rLBNSE-CfG, or DMEM by intracerebral (i.c.) injection (30 μL). The mice were euthanized on day 6 for the collection of brain tissues. Samples were harvested at a fixed time point rather than after matching animals by clinical score. This sampling design aimed to capture brain proteomic changes on day 6 after direct intracerebral (i.c.) injection, but it could not fully separate G-protein-associated effects from differences related to disease severity.

### 2.17. Proteomic Analysis of Mouse Brain Tissue

DIA proteomics was used to identify and quantify proteins in the samples. Proteins were extracted with a phenol extraction buffer containing 500 mM Tris-HCl, 50 mM EDTA, 700 mM sucrose, 100 mM KCl, 2% β-mercaptoethanol, and 1 mM PMSF; precipitated with prechilled acetone overnight at −20 °C; and dissolved in 4% SDS. Protein concentration was determined by the BCA assay, and sample quality was preliminarily assessed by SDS-PAGE. For each sample, 100 μg of protein was digested using the filter-aided sample preparation method. Proteins were reduced with 10 mM DTT at 37 °C for 2.5 h, alkylated with 50 mM IAA in the dark for 40 min, and digested with sequencing-grade trypsin at a protein-to-trypsin ratio of 50:1 (*w*/*w*) at 37 °C for 16 h. Peptides were collected and lyophilized, and a pooled peptide sample was fractionated by high-pH reversed-phase chromatography into three fractions for DDA spectral-library construction. For both DDA and DIA analyses, 2 μg of peptides was loaded onto a C18 analytical column (3 μm, 100 Å, 75 μm × 15 cm). Mobile phase A is 0.1% formic acid in water, and mobile phase B is 0.1% formic acid in 80% acetonitrile/water. Peptides are separated at a flow rate of 600 nL/min using a 120 min gradient: 5–10% B from 0 to 13 min, 10–30% B from 13 to 93 min, 30–45% B from 93 to 113 min, and 45–95% B from 113 to 114 min, followed by 95% B from 114 to 120 min. Mass spectrometric analysis was performed on an Orbitrap Fusion Lumos mass spectrometer (Thermo Fisher Scientific, Waltham, MA, USA). The spray voltage was set to 2.1 kV, the capillary temperature to 320 °C, and the S-lens RF level to 30. Full MS scans were acquired over an *m*/*z* range of 400–1200 at a resolution of 120,000 at *m*/*z* 200, with an AGC target of 4 × 10^5^ and a maximum injection time of 50 ms. MS/MS spectra are acquired at a resolution of 30,000 at *m*/*z* 200, with fragment-ion scanning beginning at *m*/*z* 110, an AGC target of 1 × 10^5^, and a maximum injection time of 50 ms. Precursor ions were fragmented by higher-energy collisional dissociation using a normalized collision energy of 32. For spectral-library construction, the three high-pH reversed-phase peptide fractions were analyzed in DDA mode. Data-dependent MS/MS acquisition was performed using a Top 20 method with a precursor isolation window of 1.6 *m*/*z* and a dynamic exclusion time of 15 s. The DDA raw files were searched against the UniProt Mus musculus protein database (release 2026_1) using Proteome Discoverer v2.1 with the Sequest HT search engine. Trypsin was specified as the digestion enzyme, allowing up to two missed cleavages. Carbamidomethylation of cysteine was set as a fixed modification, while methionine oxidation and protein N-terminal acetylation were set as variable modifications. The precursor and fragment mass tolerances were ±10 ppm and ±0.02 Da, respectively, and the peptide-level FDR and protein-level q value were controlled at ≤1%. For quantitative analysis, each biological sample was acquired once in DIA mode. Variable precursor-isolation windows covered approximately *m*/*z* 410.455–1200.838, with nominal window widths of approximately 20 Da across *m*/*z* 400–800, 40 Da across *m*/*z* 800–1000, and 50 Da across *m*/*z* 1000–1200. The DIA raw files were analyzed in Skyline against the DDA-derived spectral library. Chromatographic peak areas of unique peptides were extracted to represent relative protein abundance. Peptides of 6–25 amino acids were retained, and b, y, and p ions were used for quantification. For each peptide, 2–5 fragment ions were selected. The chromatographic extraction window was set to 5 min, and transitions with dot-product scores below 0.6 were excluded. Protein abundance values were log-transformed and normalized across LC-MS/MS runs using median-based normalization. Missing values were handled within the DIA data-processing workflow, and no additional missing-value imputation was performed before differential analysis. The normalized protein-abundance matrix was used for subsequent statistical comparisons.

### 2.18. Statistics and Data Analysis

All data are presented as the mean of three biological replicates. Experimental data are expressed as mean ± SD. Statistical analyses were performed using GraphPad Prism v8.0 and SPSS v14.0. One-way or two-way ANOVA followed by post hoc tests were used. *p* < 0.05 was considered statistically significant for all comparisons. Symbols indicate significance levels: * *p* < 0.05, ** *p* < 0.01, *** *p* < 0.001. Survival curves were created using the Kaplan–Meier method, and any differences in the survival curves were compared using the log-rank test followed by post hoc power analysis. Differentially expressed proteins were identified by statistical analysis of the protein relative-abundance matrix. *p* values were adjusted for multiple testing using FDR correction, and proteins with FDR < 0.05 were considered differentially expressed. No additional fold-change cutoff or batch correction was applied. ClusterProfiler software v4.16.0 was used for all enrichment analysis, including Gene Ontology (GO) enrichment and the Kyoto Encyclopedia of Genes and Genomes (KEGG).

## 3. Results

### 3.1. Construction of the r-pLBNSE-SfG, r-pLBNSE-CfG, and r-pLBNSE Plasmids

To directly compare the contribution of RABV G proteins from strains with different virulence backgrounds, we first engineered the pCDNA3.1-LBNSE backbone to allow for G-gene replacement (Figure 1a). We used overlap PCR to modify selected restriction sites without disrupting the recombinant genome framework. Specifically, the *EcoR*I site was mutated at nucleotide positions 1126 and 1129; the original *Pst*I site was mutated at positions 4961, 4962, and 4965; and a new *Pst*I site was introduced at positions 3324 and 3325. Sanger sequencing confirmed all designed substitutions, including GAATTC to GAGTTT, CTCAAG to CTGCAG, and CTGCAG to TTGGCG (Figure 1b–d). We then amplified the G genes from the attenuated SAD-B19 strain and the laboratory-adapted CVS11 strain using RABV-G F/R primers and inserted them into the modified r-pLBNSE backbone through *Pst*I and *BsiW*I digestion (Figure 1e). This strategy produced two recombinant full-length plasmids: r-pLBNSE-SfG, carrying the SAD-B19-G gene, and r-pLBNSE-CfG, carrying the CVS11-G gene. Sequencing of the recombinant plasmids confirmed correct insertion of the full RABV G coding sequences and the designed Flag-tagged N-terminal region (Figure 1f,g).

### 3.2. rLBNSE-CfG Shows Enhanced Neurovirulence After Direct Intracerebral (i.c.) Injection

**Figure 2:**
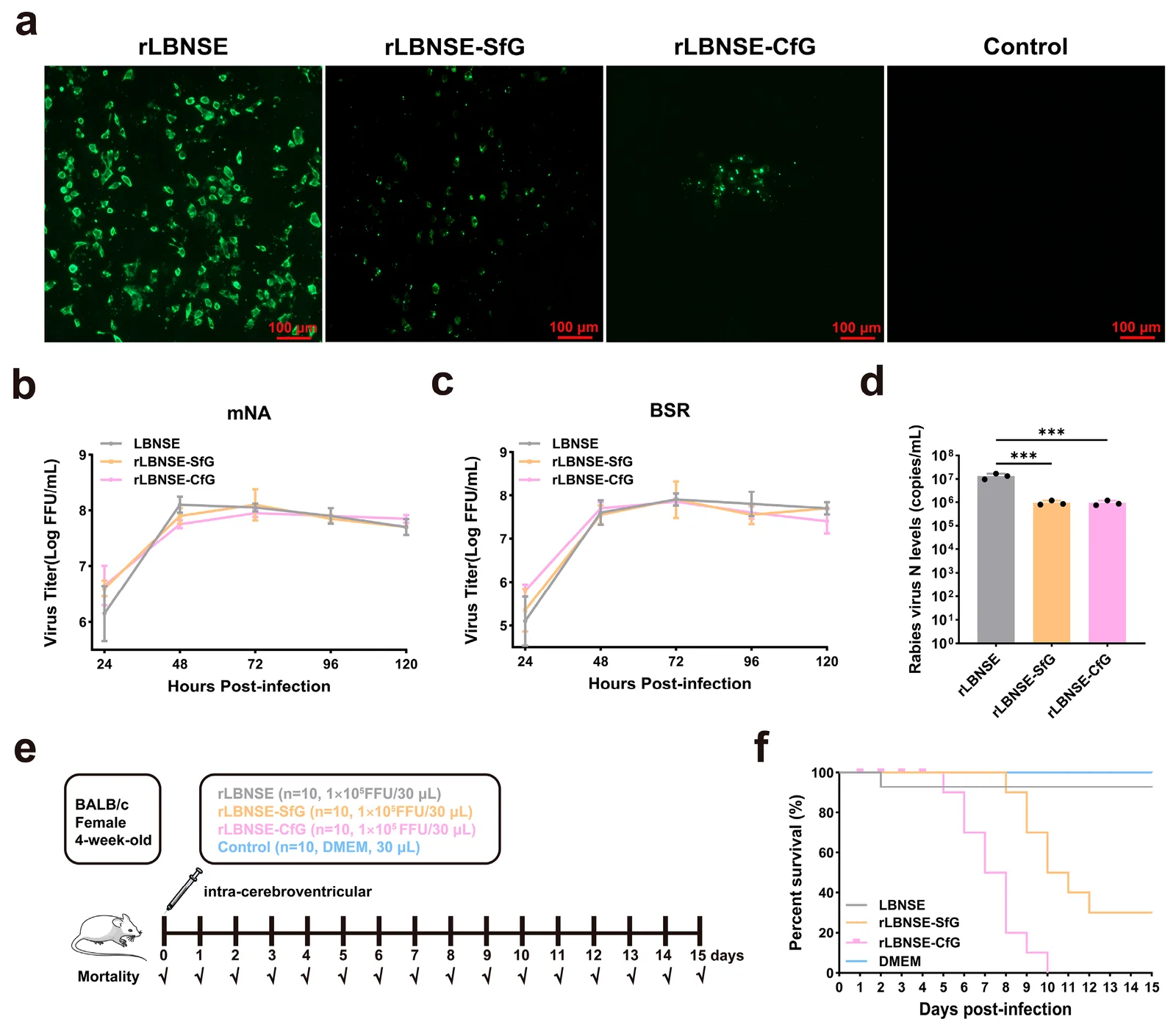
Rescue and in vitro validation of rLBNSE-SfG and rLBNSE-CfG. (**a**) Fluorescence assay (FA)-detected recombinants, where rLBNSE served as a positive control and the control group showed no fluorescence; (**b**) in vitro growth curves of rLBNSE-SfG and rLBNSE-CfG in mNA cells; (**c**) in vitro growth curves of rLBNSE-SfG and rLBNSE-CfG in BSR cells; (**d**) expression levels of the N gene in recombinants were measured by RT-PCR using N F/R primers. Copy numbers were calculated from CT values on the standard curve; (**e**) four-week-old BALB/c mice (*n* = 10 per group) were challenged with 1 × 10^5^ FFU/30 μL recombinants and parental virus by intracerebral (i.c.) injection, with DMEM used as the control; (**f**) survival rates of mice infected with the recombinants. A post hoc power analysis showed that, at α = 0.05, the power to detect the observed differences was 79.7% (rLBNSE-CfG vs. rLBNSE-SfG) and 93.8% (rLBNSE-CfG vs. rLBNSE), respectively, which is at or above the conventional 80% threshold. (**b**–**d**) are presented as the mean of three biological replicates. Experimental data are expressed as mean ± SD. One-way or two-way ANOVA followed by post hoc tests were used. *p* < 0.05 was considered statistically significant for all comparisons. Symbols indicate significance levels: *** *p* < 0.001. Survival curves were created using the Kaplan–Meier method, and any differences in the survival curves were compared via the log-rank test. The intracerebral (i.c.) challenge model evaluates neurovirulence after direct CNS exposure and does not model natural peripheral rabies transmission.

To rescue recombinants carrying G proteins from different RABV strains, we co-transfected the full-length plasmids r-pLBNSE-SfG, r-pLBNSE-CfG, and r-pLBNSE into BSR cells together with the helper plasmids pCDNA3.1-N, pCDNA3.1-P, pCDNA3.1-G, and pCDNA3.1-L. Culture supernatants collected on day 6 were used to infect BSR cells, and viral rescue was verified 48 h later by DFA using a FITC-labeled mouse anti-RABV N monoclonal antibody. Strong N-specific fluorescence was localized to the cell membrane and cytoplasm in the rLBNSE-SfG group, whereas only a few fluorescent cells appeared in the rLBNSE-CfG group; no fluorescence was seen in uninfected BSR cells (Figure 2a). These results confirmed the successful rescue of rLBNSE-SfG, rLBNSE-CfG, and the parental rLBNSE virus. To assess whether G protein substitution altered in vitro propagation, we infected BSR and mNA cells at an MOI of 0.1. All three recombinants showed comparable growth kinetics in both cell lines, with titers peaking at 72 h and exceeding 1 × 10^8^ FFU/mL (Figure 2b,c), indicating that G gene replacement did not impair overall replication efficiency in vitro. Quantitative RT-PCR of RNA extracted from infected BSR cells at 72 h showed that RABV N gene expression was significantly lower in the rLBNSE-SfG and rLBNSE-CfG groups than in the parental rLBNSE group (*p* < 0.001, Figure 2d). We next compared in vivo pathogenicity by challenging 4-week-old BALB/c mice via intracerebral (i.c.) injection with F6-generation recombinants at 1 × 10^5^ FFU/30 μL, and monitoring them for 15 days (Figure 2e). Neurological signs, including hyperexcitability, piloerection, and behavioral abnormalities, appeared in all virus-challenged groups by day 3 post-challenge. Mice challenged with rLBNSE-CfG began to die on day 5 and reached 100% mortality by day 10, whereas the rLBNSE-SfG group began to die on day 8 and reached 70% mortality by day 12. In contrast, only one mouse in the rLBNSE group died (10% mortality), and all DMEM-treated control mice survived (Figure 2f). Thus, although G protein replacement did not impair in vitro viral growth, the CVS11-derived G protein was associated with greater neurovirulence than the SAD-B19-derived G protein after direct intracerebral (i.c.) injection.

### 3.3. rLBNSE-CfG Shows Increased Early Neuronal Infectivity and Distinct G-Protein Electrophoretic Migration

**Figure 3:**
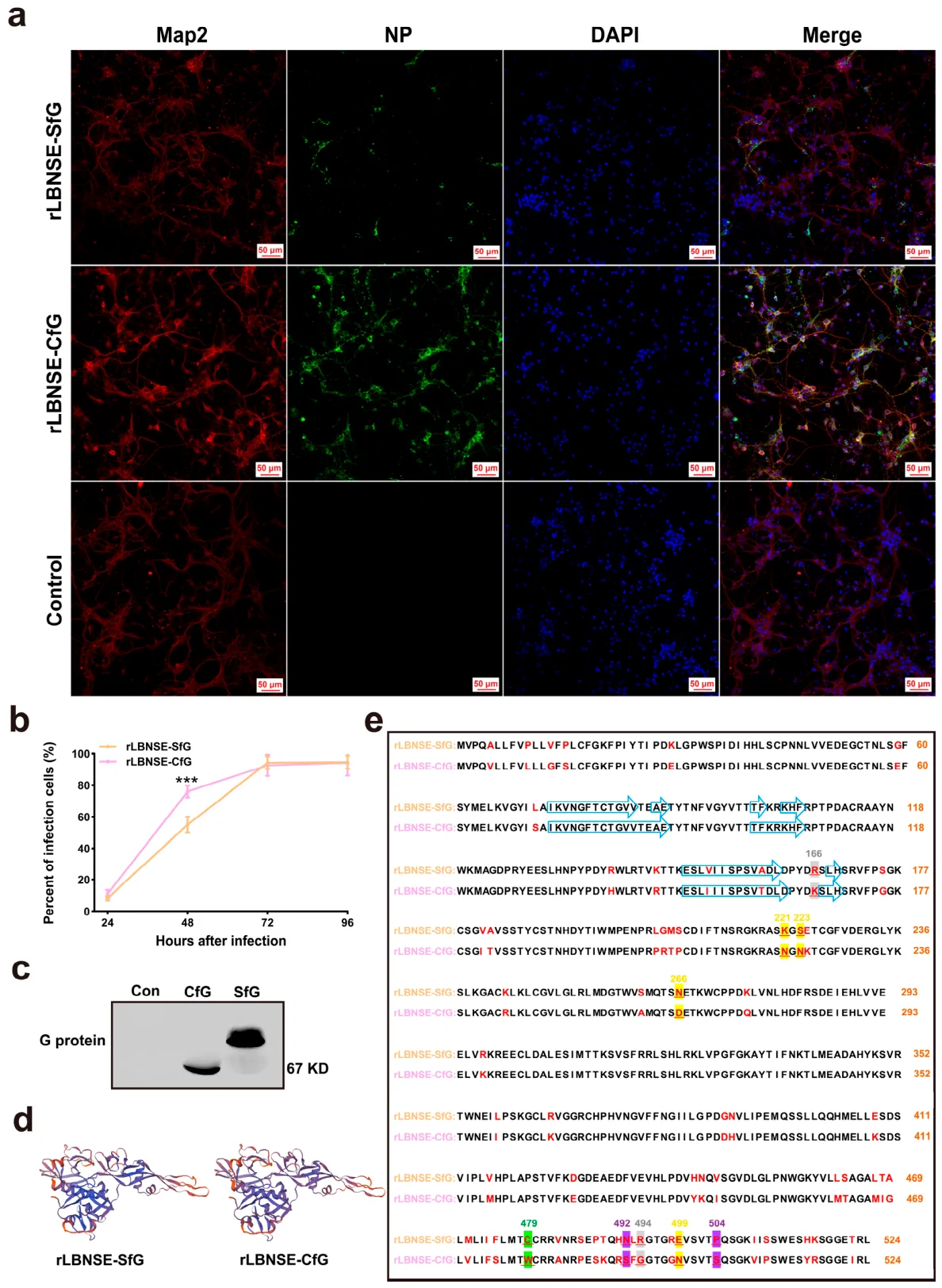
rLBNSE-CfG shows increased early neuronal infectivity and distinct G-protein electrophoretic migration. (**a**) Primary neurons from fetal mice were infected with rLBNSE-SfG or rLBNSE-CfG, and expression of neuron-specific proteins (Map2 and RABV NP) was detected. Map2 was labeled with CY3, N protein with FITC, and nuclei with DAPI; (**b**) primary neonatal mouse neurons were infected with rLBNSE-SfG or rLBNSE-CfG, and infection rate was assessed by IFA at 24, 48, 72, and 96 h. Each group had three biological replicates (*n* = 3); in each well, three fields of view were randomly selected. The entire infection experiment was independently repeated three times; (**c**) Western blot detection of rLBNSE-SfG and rLBNSE-CfG using anti-Flag antibodies; (**d**) predicted tertiary structures of the G protein from SAD-B19 and CVS11 strains generated with SWISS-MODEL; (**e**) comparison of amino acid differences between SAD-B19 and CVS11 G proteins from the NCBI database; blue arrow boxes indicate β-sheets, yellow background indicates predicted N-linked glycosylation (N), gray background indicates predicted methylation (R), green background indicates predicted S-nitrosylation (C), and purple background indicates predicted O-linked glycosylation (S, T). (**b**) presents the mean of three biological replicates. Experimental data are expressed as mean ± SD. Two-way ANOVA followed by post hoc tests were used. *p* < 0.05 was considered statistically significant for all comparisons. Symbols indicate significance levels: *** *p* < 0.001.

To compare neuronal infection by the two recombinants, primary mouse neurons were infected with rLBNSE-SfG or rLBNSE-CfG at an MOI of 0.01, and infection kinetics were assessed by DFA at multiple time points. Neuronal morphology was monitored in parallel by Map2 immunostaining. At 48 h post-infection, rLBNSE-CfG infected a significantly higher proportion of primary neurons than rLBNSE-SfG (*p* < 0.01, Figure 3a,b), indicating more rapid early neuronal infection by rLBNSE-CfG. By 72 h, both recombinants had established infection in primary neurons, and the difference in infection rate was not significant (Figure 3b). These kinetics supported the use of an MOI of 0.01 and a 72 h infection period for subsequent neuronal assays. We next verified the expression of Flag-tagged G proteins in infected neurons by Western blotting. The rLBNSE-SfG G protein migrated at a higher apparent molecular weight than the rLBNSE-CfG G protein (Figure 3c and Figure S4). Structural analysis showed that the two G proteins retained broadly similar tertiary conformations (Figure 3d), indicating that the difference in apparent molecular weight was unlikely to reflect a major alteration in overall folding. Sequence comparison identified multiple amino acid substitutions and predicted differences in putative modification sites between the two G proteins. Bioinformatic analysis predicted strain-specific differences in N-linked glycosylation, O-linked glycosylation at serine/threonine residues, arginine methylation, and cysteine S-nitrosylation sites (Figure 3e). However, the biochemical basis of the different electrophoretic migration patterns was not determined in the present study.

### 3.4. Proteomic Profiling Identifies a Downregulated Presynaptic Vesicle-Cycle Signature After rLBNSE-CfG Challenge

**Figure 4:**
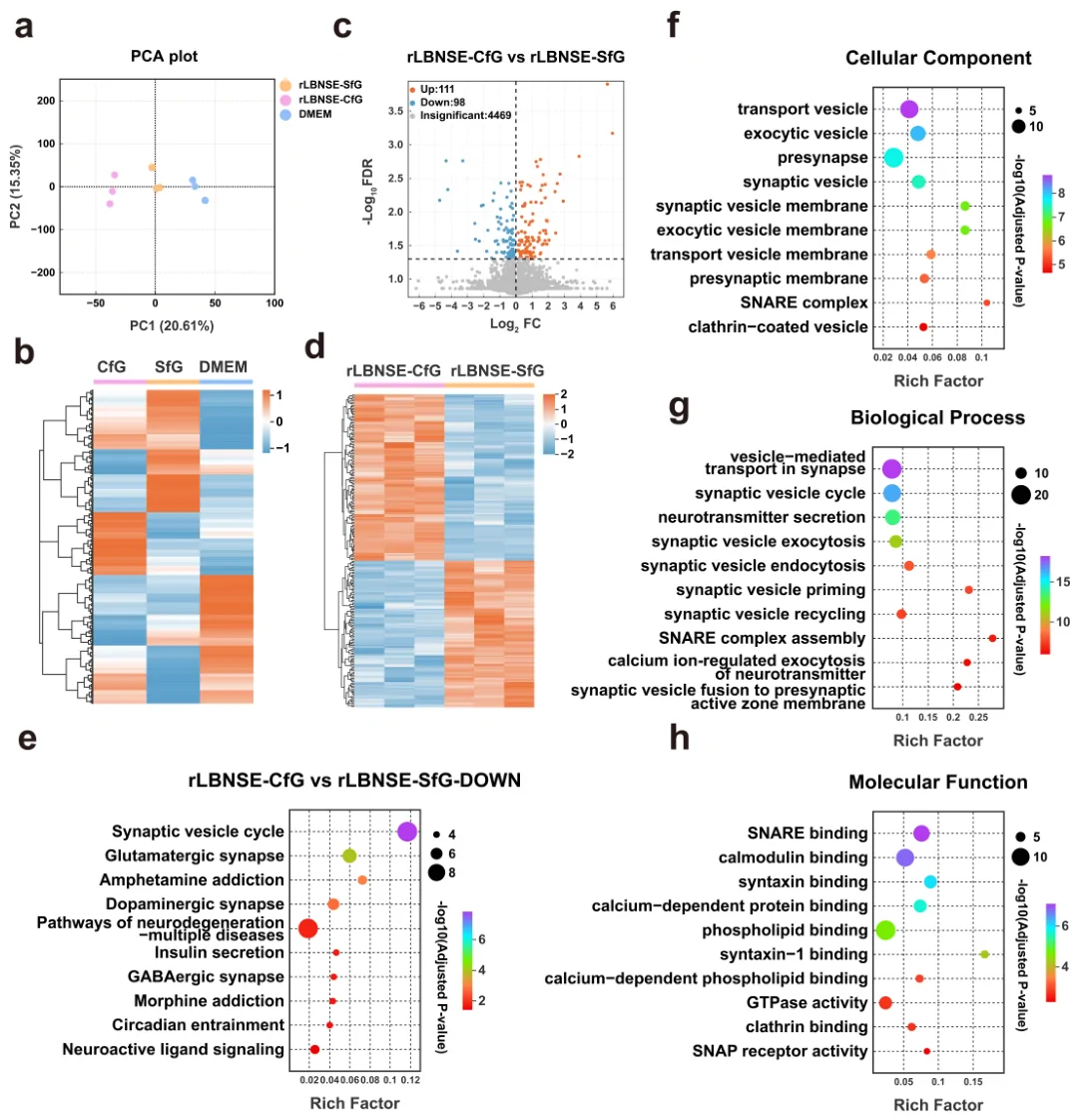
Day-6 brain proteomic remodeling after intracerebral (i.c.) rLBNSE-CfG challenge is characterized by the downregulation of proteins annotated to the synaptic vesicle cycle. (**a**) PCA plot for rLBNSE-SfG, rLBNSE-CfG, and DMEM groups; (**b**) heatmap of protein abundances across the three groups; (**c**) volcano plot of differentially expressed proteins between rLBNSE-CfG and rLBNSE-SfG; (**d**) heatmap of differentially expressed proteins between rLBNSE-CfG and rLBNSE-SfG; (**e**) KEGG enrichment analysis of downregulated proteins between rLBNSE-CfG and rLBNSE-SfG; (**f**–**h**) GO enrichment analysis of downregulated proteins, covering (**f**) cellular components, (**g**) biological processes, and (**h**) molecular functions.

To define host brain responses associated with the two recombinant G proteins, mouse brain tissues were collected on day 6 post-challenge for quantitative proteomic analysis. The abundance distributions, density curves, mass-error distribution, and sample correlations support acceptable quantitative consistency for downstream analysis (Figure S1). PCA and hierarchical clustering showed group-level separation among the rLBNSE-SfG, rLBNSE-CfG, and DMEM samples, indicating distinct bulk-brain proteomic profiles at the day-6 sampling time point (Figure 4a). Consistently, clustering of global protein abundance distinguished the rLBNSE-SfG, rLBNSE-CfG, and DMEM groups (Figure 4b). Direct comparison of the rLBNSE-CfG with rLBNSE-SfG groups identified 209 differentially expressed proteins, including 111 upregulated and 98 downregulated proteins in the rLBNSE-CfG group (FDR < 0.05, Figure 4c). Heatmap analysis further showed a clear separation of these differentially expressed proteins between the two recombinants (Figure 4d). Functional enrichment of the rLBNSE-CfG downregulated proteins revealed a presynaptic signature. KEGG pathway analysis independently identified the synaptic vesicle cycle as the most enriched pathway among the downregulated proteins (Figure 4e), linking the proteomic changes to presynaptic vesicle trafficking and release. GO enrichment further refined this signature. Biological process terms were dominated by vesicle-mediated transport in the synapse, synaptic vesicle cycle, neurotransmitter transport, neurotransmitter secretion, signal release from the synapse, synaptic vesicle exocytosis, and calcium-ion-regulated exocytosis (Figure 4f). Cellular component terms were enriched for presynapse and synaptic vesicle membrane (Figure 4g), whereas molecular function terms included SNARE binding and syntaxin binding (Figure 4h). In contrast, proteins upregulated in the rLBNSE-CfG group were enriched in pathways and functional categories associated with cytoskeletal remodeling, immune-related protein processing, and cellular stress responses. KEGG analysis showed the cytoskeleton in muscle cells, antigen processing and presentation, glycolysis/gluconeogenesis, focal adhesion, ECM–receptor interaction, and protein processing in the endoplasmic reticulum (Figure S2a). GO enrichment was associated with the regulation of peptidase and endopeptidase activity, proteolysis, apoptotic signaling, the cortical cytoskeleton, myofibril, extracellular matrix structural constituent, actin filament binding, cytoskeletal structural activity, and antioxidant activity (Figure S2b–d). Comprehensive proteomic analysis revealed that rLBNSE-CfG was associated with the coordinated downregulation of proteins annotated to the presynaptic vesicle-cycle, SNARE-related, and neurotransmitter-secretion pathways, together with the upregulation of proteins linked to cytoskeletal organization, antigen processing, protein quality control, and metabolic stress adaptation. Because the samples were collected at a fixed post-challenge time point, these proteomic differences may reflect a combination of G-protein-associated effects and differences in disease severity, neuronal injury, inflammation, or cellular composition.

### 3.5. rLBNSE-CfG Is Associated with Reduced Vamp2 Abundance in Primary Neurons

**Figure 5:**
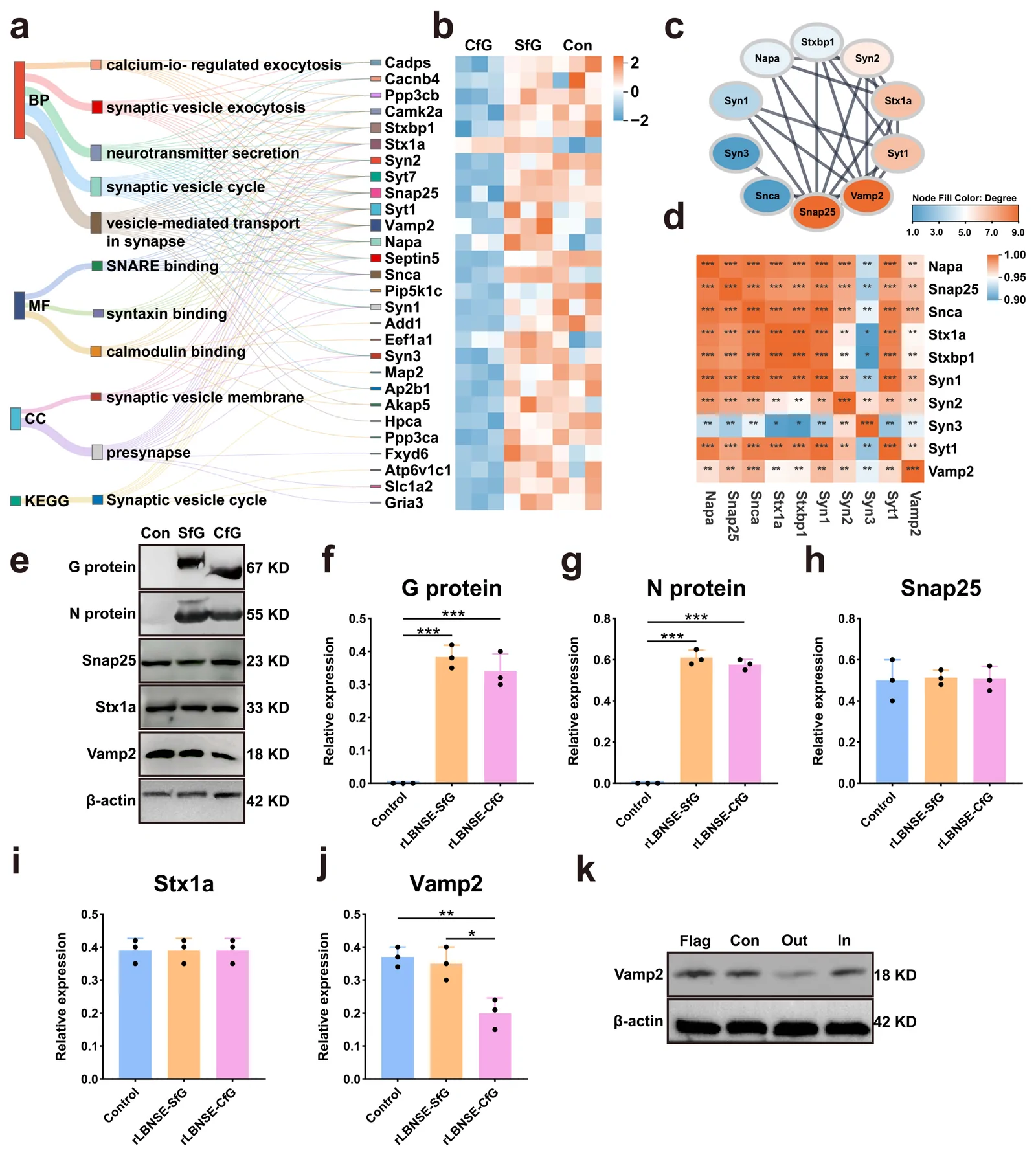
Reduced abundance of selected SNARE-associated proteins in association with rLBNSE-CfG infection. (**a**) Sankey plot showing the presynaptic vesicle-cycle-related protein module constructed from representative GO and KEGG terms. (**b**) Heatmap showing the abundance of synaptic vesicle-cycle-associated downregulated proteins across rLBNSE-SfG, rLBNSE-CfG, and DMEM groups. (**c**) STRING protein association network of synaptic vesicle-cycle-associated downregulated proteins constructed using a high confidence threshold (0.9). Node color represents network degree. (**d**) Pearson correlation heatmap showing abundance-based correlations among selected synaptic vesicle-cycle-associated proteins; (**e**) Western blot of G protein, N protein, Snap25, Stx1a, and Vamp2 in control, rLBNSE-SfG, and rLBNSE-CfG groups. (**f**–**j**) Relative expression of proteins. (**k**) The plasmids pCDNA3.1-CVS11-G-outside and pCDNA3.1-CVS11-G-inside were separately transfected into mouse primary neurons. Protein was extracted 72 h after transfection, and target protein expression was detected by Western blot using a Vamp2 antibody. (**e**,**k**) are presented as the mean of three biological replicates. Experimental data are expressed as mean ± SD (**f**–**j**). One-way ANOVA followed by post hoc tests was used. *p* < 0.05 was considered statistically significant for all comparisons. Symbols indicate significance levels: * *p* < 0.05, ** *p* < 0.01, *** *p* < 0.001.

To refine the presynaptic proteomic signature associated with rLBNSE-CfG infection, we extracted 28 proteins annotated to the top synaptic vesicle-cycle-related GO terms and integrated their functional annotations (Figure 5a). These proteins showed a coordinated reduction in the rLBNSE-CfG group compared with the rLBNSE-SfG and DMEM groups (Figure 5b). A high-confidence STRING network constructed with a confidence score of 0.9 revealed a functionally connected synaptic vesicle-associated protein module that prioritized Vamp2, Stx1a, and Snap25 as core SNARE-related proteins linking vesicle fusion, presynaptic membrane organization and neurotransmitter release (Figure 5c). Abundance-based correlation analysis further showed positive associations among members of this module (Figure 5d). Western blotting confirmed comparable expression of RABV G and N proteins in rLBNSE-SfG- and rLBNSE-CfG-infected neurons (Figure 5e–g and Figure S5). Among the selected SNARE proteins, Vamp2 was significantly reduced in the rLBNSE-CfG group, whereas Stx1a and Snap25 showed no significant differences between groups (Figure 5e,h–j). To test whether this effect mapped to a specific region of the CVS11 G protein, primary mouse neurons were transfected with plasmids encoding either the extracellular or intracellular domain of CVS11-G. To test whether this effect mapped to a specific region of the CVS11 G protein, primary mouse neurons were transfected with plasmids encoding either the extracellular or intracellular domain of CVS11-G. Expression of the extracellular domain of the CVS11 G protein reduced Vamp2 protein levels, whereas the intracellular-domain construct and Flag-vector control did not produce this effect (Figure 5k and Figure S6). To further validate the domain-transfection experiment, we performed immunofluorescence staining with anti-Flag and Map2 antibodies. Flag signals from both the extracellular- and intracellular-domain constructs were detected in Map2-positive neuronal cultures, confirming expression and neuronal localization of the isolated CVS11-G fragments (Figure S3a). RT-qPCR showed that *Vamp2* mRNA expression was significantly reduced in the extracellular-domain group compared with the Flag-vector control, untreated control, and intracellular-domain groups (*p* < 0.05; Figure S3b). In contrast, cell viability did not differ significantly among the four groups (Figure S3c), indicating that the reduction in Vamp2 expression was unlikely to reflect overt transfection-associated cytotoxicity. Together, these findings support an association between the CVS11-G extracellular-domain construct and reduced Vamp2 expression in primary neurons and suggest that this effect may involve a transcriptional component. Together, these data indicate that reduced Vamp2 abundance is part of a presynaptic vesicle-cycle/SNARE-related proteomic signature associated with rLBNSE-CfG challenge. The domain-fragment transfection assay further supports a potential link between CVS11-G and Vamp2 abundance in primary neurons, although the observed brain proteomic differences may also reflect variation in disease severity and cellular composition at the sampling time point.

## 4. Discussion

Rabies virus (RABV) is one of the most lethal known zoonotic pathogens; once clinical symptoms appear, the case-fatality rate approaches 100%, and there are no effective specific clinical treatments. The G protein, the sole envelope glycoprotein, is both the primary protective antigen that elicits host neutralizing antibodies and a core virulence determinant of neurotropism and pathogenicity. However, the molecular mechanisms by which G proteins from different strains remodel the neuronal proteome, disrupt synaptic transmission, and cause neurological dysfunction remain incompletely understood. In this study, we constructed recombinant RABVs carrying G proteins of differing virulence and combined primary neuronal infection models, in vivo neurovirulence assays, and quantitative proteomics. The results showed that rLBNSE-CfG challenge was associated with reduced Vamp2 abundance and altered abundance of proteins associated with presynaptic vesicle-cycle and SNARE-related pathways. These findings identify an rLBNSE-CfG-associated presynaptic proteomic signature rather than demonstrating direct or specific regulation of these proteins by CVS11-derived G in vivo. Because bulk brain tissues were collected at a fixed day-6 time point rather than matched according to clinical severity, the observed proteomic differences may also reflect disease progression-related changes, including neuronal injury, inflammation, or altered cellular composition. In addition, intracerebral (i.c.) injection bypasses peripheral invasion, neuromuscular-junction infection, and retrograde axonal transport. Therefore, the observed mortality differences primarily reflect neurovirulence after direct central nervous system exposure rather than the complete course of natural peripheral infection. This experimental approach is consistent with previous studies that used intracerebral (i.c.) challenge to compare the central nervous system effects of RABV G-protein variation [18,35].

In this study, we applied reverse genetics techniques [36,37]. By precisely modifying restriction sites in the pCDNA3.1-LBNSE vector, we constructed recombinant RABVs carrying the G protein from the attenuated strain SAD-B19 (rLBNSE-SfG) and the G protein from the virulent strain CVS11 (rLBNSE-CfG). We introduced three site-directed mutations to alter the vector’s restriction sites and fully validated these changes by sequencing, ensuring that the recombinants shared an identical backbone. Although the Flag tag design has been functionally validated in our group’s previous study and in this study through replication kinetics, membrane localization, and neuronal infection experiments [38], we cannot completely rule out subtle effects of the tag on the fine conformation of the G protein. Future structural studies comparing tagged and untagged G proteins will provide more definitive confirmation of this. In summary, the design used in this study ensured that the phenotypic differences observed in subsequent experiments were attributable to the G protein sequence itself rather than to scaffold mutations. Notably, the rescue success rate of rLBNSE-CfG was lower than those of rLBNSE-SfG and rLBNSE, a finding consistent with the high neurotropic virulence of the CVS11 strain [25]. Host cells experienced stronger virulence effects during rescue with recombinants carrying the CVS11 G protein, indirectly confirming the high virulence of the CVS11 G protein. The G gene is one of the most variable genes in the RABV genome [39], and the extensive sequence differences between the two strains provide an important basis for subsequent functional comparisons. Western blot analysis showed that the rLBNSE-SfG G protein migrated at a higher apparent molecular weight than the rLBNSE-CfG G protein. However, electrophoretic migration alone cannot identify the biochemical basis of this difference. Glycosylation is an important post-translational modification of viral envelope proteins, and variation in N-glycosylation sites among RABV G proteins has been associated with differences in viral budding efficiency and antigenic properties [40]. S-nitrosylation can alter protein conformation, stability, subcellular localization, and activity and is implicated in innate immune signaling [41]. In addition, strain-specific amino acid substitutions in RABV G can also alter protein structure and properties related to virulence [18]. However, these known biological roles do not demonstrate that the migration difference observed here arises from glycosylation, methylation, S-nitrosylation, or any other specific modification. Because PNGase F or Endo H digestion, site-directed mutagenesis, glycoproteomic analysis, and LC-MS/MS confirmation of modified peptides were not performed, no specific post-translational modification can be assigned to either G protein. Thus, the biochemical basis of the observed electrophoretic migration difference remains undetermined.

In vitro replication kinetics experiments showed that the proliferation patterns of rLBNSE-SfG and rLBNSE-CfG in BSR and mNA cells did not differ significantly from those of the parental virus rLBNSE (*p* > 0.05), with both reaching peak titers (>1 × 10^8^ FFU/mL) 72 h post-infection. The peak titers of the three recombinants were comparable, exceeding 1 × 10^8^ FFU/mL, as determined by the fluorescence focus assay (FFA). However, RT-qPCR revealed that the N gene expression levels of both rLBNSE-SfG and rLBNSE-CfG were significantly lower than those of the parental strain rLBNSE (*p* < 0.001). This apparent discrepancy reflects differences in the parameters measured by the two assay methods. FFA quantifies infectious viral particles capable of establishing infection, while RT-qPCR measures total viral RNA copies, including RNA from non-infectious or defective particles. The G protein substitution may have altered the ratio of particles to FFUs, resulting in reduced transcriptional output per infectious unit without compromising the overall infectivity. Notably, reduced N gene expression was observed in both recombinant viruses. Therefore, this effect can be attributed to the G protein substitution itself rather than to a specific G protein sequence, and thus it cannot explain the difference in virulence between rLBNSE-CfG and rLBNSE-SfG. This result indicated that replacing the G protein did not affect the virus’s basic replication capacity, effectively ruling out differences in replication efficiency as a confounding factor in subsequent pathogenicity studies and ensuring the scientific validity of the virulence comparison experiments. In the primary neuron infection model, the neuronal infection rate of rLBNSE-CfG at 48 h post-infection was significantly higher than that of rLBNSE-SfG (*p* < 0.01), whereas the difference disappeared by 72 h. This early difference in infection efficiency suggests that the CVS11 G protein has a greater capacity for early neuronal invasion, which may relate to the residue at position 474 of the G protein’s cytoplasmic tail (the SAD-B19 and CVS11 strains carry Ile and Phe at this position, respectively), and previous studies have shown that Phe_474_ enhances CVS11 internalization efficiency in neurons [21]. This inferred structure–function relationship provides a plausible molecular explanation for the difference in early infection efficiency between the two recombinants and further confirms the critical regulatory role of the G protein in neuronal infection in vitro. The intracerebral (i.c.) injection challenge experiment in mice demonstrated a clear virulence gradient among the three recombinants, rLBNSE-CfG (100% mortality, day 10) > rLBNSE-SfG (70% mortality, day 12) > rLBNSE (10% mortality), rather than following natural peripheral exposure. One mouse in the rLBNSE group died on the second day after challenge. This early death occurred before neurological symptoms appeared in any group, which is inconsistent with the expected progression of rabies-virus-induced encephalitis. However, no necropsy, histopathological examination, or viral antigen testing was performed on this animal, preventing determination of the cause of death. The remaining nine mice in the rLBNSE group survived until the end of the 15-day observation period. This virulence gradient aligns closely with previous reports of high neurotropism in the CVS11 strain [20]. Different RABV strains show variable virulence because of differences in G-protein sequences. Amino acid residues closely associated with pathogenicity include positions 333, 194, 37, 242, 255, and 268 [18,19,42]. We found that the 15th residue of the CVS11 G protein is Ser, whereas that for SAD-B19 is Pro. Given evidence that the G (S15R) mutation reduced RABV virulence in Vero cells [43], we speculate that the difference at position 15 may contribute to the lethality difference between the two recombinants; however, this hypothesis requires validation by site-directed mutagenesis.

Quantitative proteomic analysis identified coordinated changes in the brain proteome following rLBNSE-CfG challenge. PCA and hierarchical clustering distinguished proteomic profiles associated with different G-protein sources. GO and KEGG enrichment analyses of the 209 differentially expressed proteins consistently identified downregulation of proteins annotated to the synaptic vesicle cycle. This pathway, which encompasses vesicle biogenesis, transport, docking, priming, fusion, and endocytosis, is central to presynaptic signaling [44]. Within this enriched protein set, Western blotting confirmed a significant reduction in Vamp2 abundance in the rLBNSE-CfG group (*p* < 0.01), while Snap25 and Stx1a were unchanged. Expression of the CVS11-G extracellular-domain construct also correlated with reduced Vamp2 abundance in primary neurons. The SNARE complex consists of the presynaptic membrane t-SNAREs Snap25 and Stx1a and the vesicle-associated v-SNARE Vamp2; together they form the core molecular machinery responsible for synaptic vesicle fusion and neurotransmitter release [45]. Genetic knockout and electrophysiological studies show that complete loss or severe disruption of Vamp2 markedly impairs synaptic vesicle exocytosis and Ca^2+^-triggered membrane fusion. These experiments define the essential physiological role of Vamp2 but do not determine the functional consequences of the partial reduction in Vamp2 abundance observed here during RABV infection. Likewise, although SNARE dysfunction has been linked to reduced neurotransmitter release, impaired synaptic transmission, and disruption of neuronal network activity in other systems, we did not directly measure those outcomes in the present study [46]. Altered Vamp2 levels have been reported in several neurological disorders. Vamp2 has been studied as a cerebrospinal fluid biomarker linked to cognitive impairment in Down syndrome and Alzheimer’s disease [47], and its concentration has been reported to differ between early and late stages of Alzheimer’s disease [48]. Insufficient presynaptic neurotransmitter release has been proposed to contribute to certain manifestations of Huntington’s disease [49]. In addition, tetanus and botulinum neurotoxins block neurotransmitter release by proteolytically cleaving Vamp2 [50]. These findings underscore Vamp2’s broader role in neurological function but do not demonstrate that the reduction we observed in the present RABV model produces comparable physiological effects. Previous studies show that neurotropic viruses, including herpes simplex virus and West Nile virus, can interfere with synaptic transmission [51,52]. Our earlier work demonstrated that siRNA-mediated depletion of Snap25 altered RABV RNA and protein abundance and reduced the infectivity of progeny viral particles [38]. However, Snap25 and Vamp2 are distinct SNARE components, and the viral phenotypes reported in that study did not directly assess synaptic vesicle release. Therefore, those findings cannot serve as functional validation of the present Vamp2 result. The current data support an association among rLBNSE-CfG challenge, reduced Vamp2 abundance, and a presynaptic vesicle-cycle/SNARE-related proteomic signature. They do not demonstrate impaired synaptic vesicle fusion, reduced neurotransmitter secretion, altered synaptic transmission, or a Vamp2-dependent mechanism of neurovirulence. The functional significance of reduced Vamp2 abundance remains unknown in this experimental system. Electrophysiological recordings, FM-dye uptake/release assays, synaptophysin-pHluorin imaging, direct neurotransmitter measurements, and Vamp2 overexpression or rescue experiments are required to determine whether the observed change in abundance affected presynaptic function.

Domain-transfection analysis in primary neurons further suggests that the extracellular domain of the CVS11 G protein contributes to the regulation of Vamp2 abundance, whereas the intracellular domain alone does not reproduce this effect. In the context of the full-length G protein, CVS11-G expression was associated with reduced Vamp2 protein abundance, and the domain-transfection experiment further narrowed this effect to the extracellular domain. However, these results do not exclude potential indirect contributions of the intracellular domain or synergistic interactions between different regions of the full-length G protein. The G protein extracellular domain serves as the primary interface for virus–host receptor interactions and is a major target for neutralizing antibodies [35]. It mediates viral entry by binding to neuronal surface receptors such as nicotinic acetylcholine receptors, the neural cell adhesion molecule NCAM, and the p75 neurotrophin receptor [53,54]. Previous studies have mainly focused on its role in viral entry; however, its potential involvement in regulating host synaptic protein expression after viral entry remains poorly understood. In this study, isolated extracellular- and intracellular-domain fragments of CVS11 G were expressed in primary neurons to further investigate the region responsible for Vamp2 regulation. Immunofluorescence analysis confirmed the expression and distribution of both CVS11-G extracellular- and intracellular-domain constructs in Map2-positive primary neurons, supporting the validity of the domain-transfection approach. CCK-8 analysis showed no evident reduction in cell viability among the different transfection groups, suggesting that reduced cell viability was unlikely to explain the Vamp2 reduction. These findings suggest that the extracellular domain of CVS11 G is sufficient to reproduce part of the Vamp2 regulatory effect observed with the full-length G protein. Nevertheless, because the extracellular-domain fragment lacks a transmembrane region and does not fully reproduce the structural context of native full-length G protein, its biological behavior may differ from that of the intact viral glycoprotein. The molecular mechanism underlying CVS11-G-mediated reduction in Vamp2 abundance remains unresolved. The present RT-qPCR analysis showed reduced *Vamp2* transcript abundance, suggesting that transcriptional regulation may contribute to the reduction in Vamp2 protein levels. However, whether this represents direct transcriptional regulation or a secondary cellular response requires further investigation. Altered protein stability, cellular stress responses, or indirect signaling effects may also contribute to Vamp2 reduction, although these possibilities were not directly examined in the present study. Although proteins involved in endoplasmic reticulum protein processing were enriched among the upregulated proteins, this pathway-level observation does not establish proteasomal or lysosomal degradation of Vamp2. Future studies incorporating receptor-blocking assays, protein-stability measurements, proteasome or lysosome inhibition, and full-length G-protein mutants will be required to clarify the molecular mechanism underlying CVS11-G-associated Vamp2 regulation.

## 5. Conclusions

In summary, CVS11-derived RABV G was associated with increased neurovirulence after direct CNS (i.c.) injection, reduced Vamp2 abundance, and coordinated alterations in presynaptic vesicle-cycle and SNARE-related proteins. Domain-transfection experiments further associated the extracellular domain of CVS11 G with reduced Vamp2 expression in primary neurons, while immunofluorescence validation confirmed the expression and neuronal distribution of the isolated CVS11-G domain constructs without evident cytotoxic effects. These findings identify a candidate presynaptic proteomic signature associated with strain-specific RABV G variation, although bulk-brain proteomic changes may partly reflect differences in disease progression rather than direct G-specific regulation. Because presynaptic vesicle fusion, neurotransmitter release, and synaptic transmission were not directly assessed, the functional consequences of reduced Vamp2 abundance remain to be determined in this RABV infection model.

## Figures and Tables

**Table 1:** Primers and restriction sites used in this study.

Primer	Nucleotide Sequence (5′-3′)	Restriction Site
RV-G-F ^1^	TCAAAAGACTGCAGGAAAGATG	/
RV-G-R ^1^	AGATGACCCAGCACTTTATAAG	/
Fragment-1-F ^2^	CCGGAATTCCTATAGTCACGCTTAACAACCAGATC	*EcoR*I
Fragment-1-R ^2^	CCCTTTCCCGAAAAACTCCTCTCCCAGATAGCCC	/
Fragment-2-F ^3^	GGAGAGGAGTTTTTCGGGAAAGGGACATTTGAAAGAAGA	/
Fragment-2-R ^3^	AGGAACCATCTTTCCTGCAGTCTTTTGAGGGATGTTAATAGTTTTT	/
Fragment-3-F ^4^	CAAAAGACTGCAGGAAAGATGGTTCCTCAGGCTCTCC	/
Fragment-3-R ^4^	CTAGCTAGCAAATCGCCAAATCGTACG	*Nhe*I
rLBNSE-SfG-F ^5^	AACTGCAGATGGTTCCTCAGGCTCTCCTGTTTGTACCCCTTCTGGTTTTTCCATTGTGTTTTGGGGATTACAAGGACGACGATGACAAGAAATTCCCTATTTACAC	PstI
rLBNSE-SfG-R ^5^	AACTGCAGTTACAGTCTGGTCTCACCCCC	BsiWI
rLBNSE-CfG-F ^6^	AACTGCAGATGGTTCCTCAGGTTCTTTTGTTTGTACCCCTTCTGGGTTTTTCGTTGTGTTTCGGGGATTACAAGGACGACGATGACAAGAAGTTCCCCATTTACAC	PstI
rLBNSE-CfG-R ^6^	AACGTACGTCACAGTCTGATCTCACCTCC	*BsiW*I
RV-N-F ^7^	TAGCACTGGCAGATGATGGA	/
RV-N-R ^7^	TTTAGAAACTCGGCGAATGA	/
Vamp2-F ^8^	CAAGTGCAGCCAAGCTCAAG	/
Vamp2-R ^8^	CAGGGATTTAAGTGCTGAAGTAAAC	/
Gapdh-F ^9^	AACTTTGGCATTGTGGAAGG	/
Gapdh-R ^9^	ACACATTGGGGGTAGGAACA	/

^1^ RV-G-F/R amplified the RABV G gene; ^2^ Fragment-1-F/R amplified the pCDNA3.1-LBNSE N gene (*EcoR*I site underlined); ^3^ Fragment-2-F/R amplified the pCDNA3.1-LBNSE N, P, and M genes; ^4^ Fragment-3-F/R amplified the pCDNA3.1-LBNSE G gene (*Nhe*I site underlined); ^5^ rLBNSE-SfG-F/R amplified the SAD-B19 strain G gene, the sequence encoding the Flag tag is underlined; ^6^ rLBNSE-CfG-F/R amplified the CVS11 strain G gene, the sequence encoding the Flag tag is underlined. The Flag tag was inserted after the predicted signal peptide sequence and before the mature G ectodomain. Both rLBNSE-SfG-F/R and rLBNSE-CfG-F/R included the signal peptide and Flag tags. ^7^ RV-N-F/R were used for RT-qPCR amplification of the RABV N gene. ^8^ Vamp2-F/R were used for RT-qPCR amplification of the *Vamp2* gene. ^9^ Gapdh-F/R were used for normalization.

## Data Availability

The original mass spectrometry raw data generated in this study are openly available in Zenodo at https://doi.org/10.5281/zenodo.22129353. Other data supporting the findings of this study are available within the article and its Supplementary Materials.

## Supplementary Materials

The following supporting information can be downloaded at https://www.mdpi.com/article/10.3390/vetsci13090919/s1. Figure S1. Quality control evaluation of the quantitative proteomic dataset; Figure S2. GO and KEGG enrichment analysis of upregulated proteins. Figure S3. Validation of CVS11-G domain expression in primary neurons and its association with Vamp2 expression. The corresponding original uncropped Western blot images are provided in Supplementary Figures S4–S6. Figure S4. Western blot detection of rLBNSE-SfG and rLBNSE-CfG using anti-Flag antibodies. Figure S5. Western blot of G protein, N protein, Snap25, Stx1a, and Vamp2 in Control, rLBNSE-SfG, and rLBNSE-CfG groups. Figure S6. CVS11 G Protein Extracellular Domain Regulates Vamp2 Expression in Primary Mouse Neurons.

## Abbreviations

The following abbreviations are used in this manuscript:

BHK-21	Baby Hamster Kidney-21 Cells
BSA	Bovine serum albumin
CNS	Central nervous system
DAPI	4′6-diamidino-2-phenylindole
DEP	Differentially expressed protein
DFA	Direct fluorescent antibody assay
DIA	Data-independent acquisition
DMEM	Dulbecco’s modified Eagle medium
FASP	Filter-aided sample preparation
FBS	Fetal bovine serum
FFU	Fluorescence focus unit
GO	Gene Ontology
HRP	Horseradish peroxidase
i.c.	Intracerebral
KEGG	Kyoto Encyclopedia of Genes and Genomes
Map2	Microtubule-associated protein 2
mNA	Mouse neuroblastoma cell
MOI	Multiplicity of infection
PBS	Phosphate-buffered saline
PBST	Phosphate-buffered saline with Tween-20
PVDF	Polyvinylidene difluoride
RABV	Rabies virus
RT-qPCR	Reverse-transcription quantitative PCR
SNARE	Soluble N-ethylmaleimide-sensitive factor attachment protein receptor
Vamp2	Vesicle-associated membrane protein 2
