# Supplementary material for: Rabies Virus Glycoprotein G Variation Is Associated with Neurovirulence and Reduced Vamp2 Abundance in a Mouse Model of Direct CNS Infection
Source: Vet Sci. 2026 Sep 7;13(9):919. doi: 10.3390/vetsci13090919 (PMC13611883; doi:10.3390/vetsci13090919)
Supplement: Supplementary file 1 [file vetsci-13-00919-s001.zip › vetsci-4407356-supplementary.pdf]

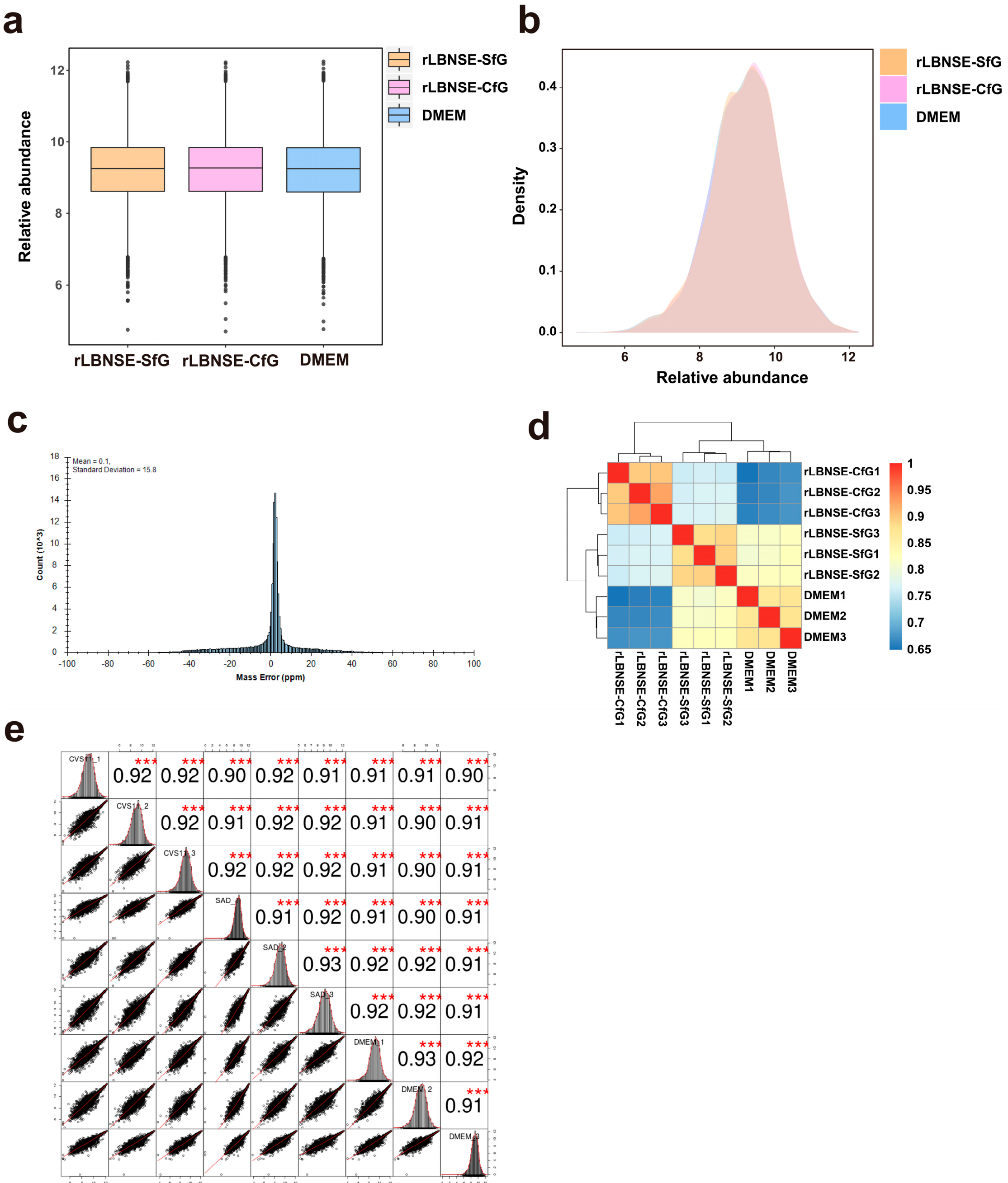

Figure S1. Quality control evaluation of the quantitative proteomic dataset. (**a**) Boxplot showing the distribution of protein relative abundances across the 3 groups; (**b**) Density plot showing the overall abundance distribution of quantified proteins; (**c**) Mass error distribution of identified peptides; (**d**) Pearson correlation heatmap showing sample-level reproducibility among biological replicates; (**e**) Pairwise scatterplot matrix showing correlations among all samples. High inter-sample correlations and comparable abundance distributions indicate that the proteomic dataset exhibited good quantitative stability and reproducibility.

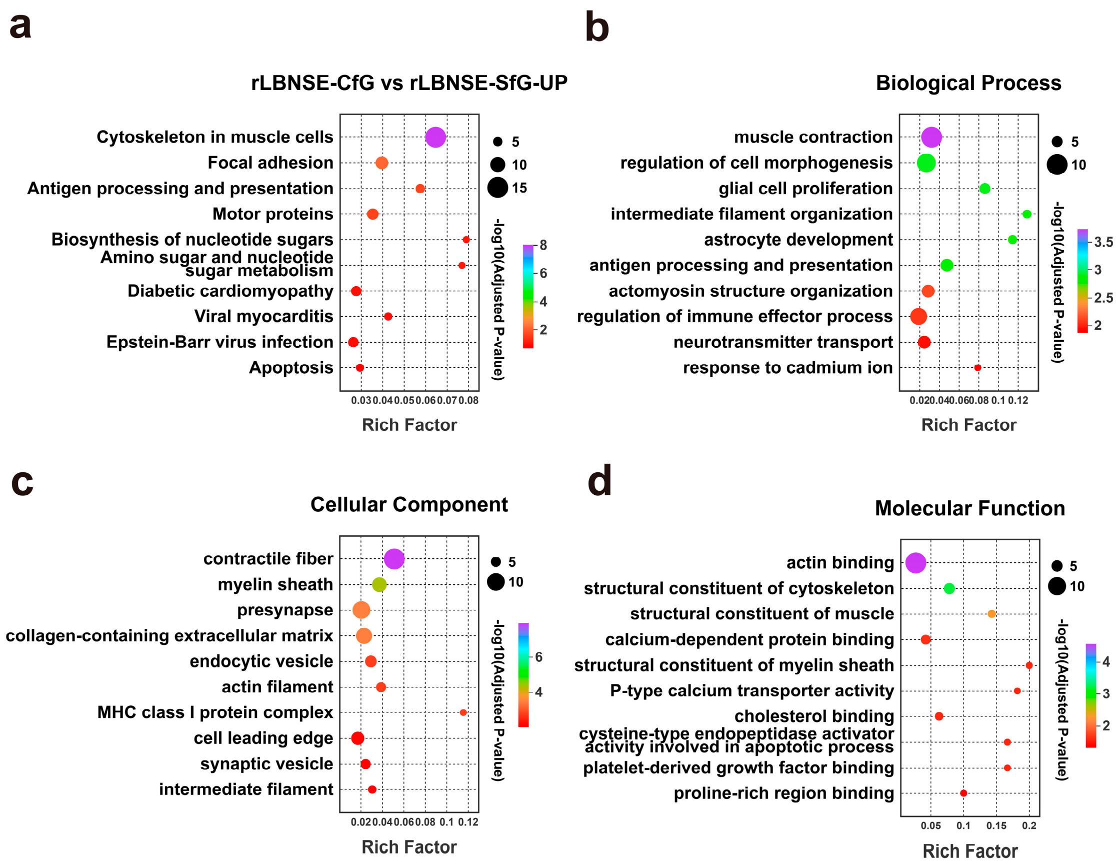

Figure S2. GO and KEGG enrichment analysis of upregulated proteins. (**a**) KEGG enrichment analysis of upregulated proteins; (**b-d**) GO enrichment analysis of upregulated proteins, covering (**b**) biological process, (**c**) cellular component, and (**d**) molecular function.

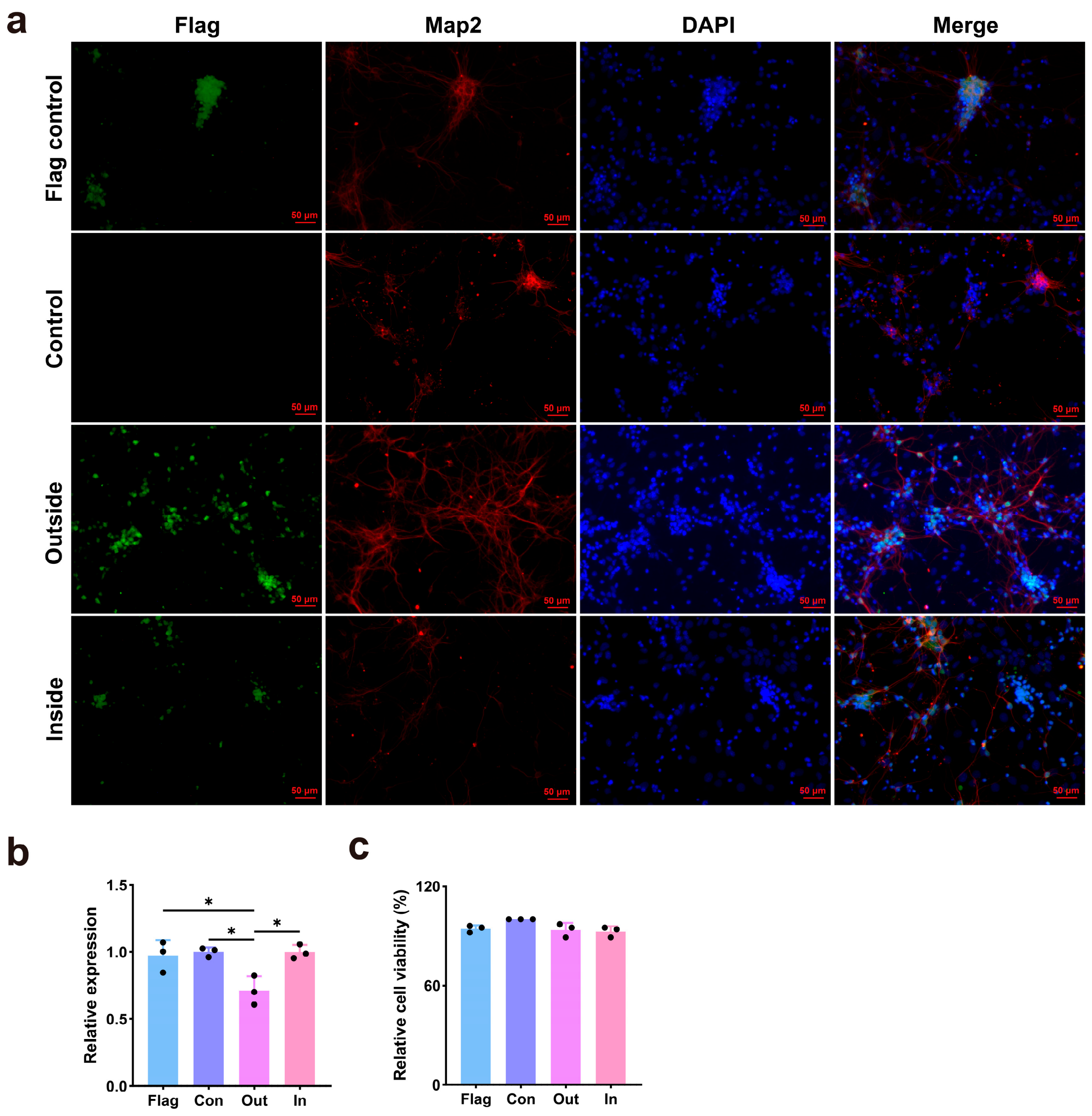

Figure S3. Validation of CVS11-G domain expression in primary neurons and its association with Vamp2 expression. (**a**) Immunofluorescence analysis of primary mouse neurons in the Flag-vector control (Flag), untreated control (Control), Flag-tagged CVS11-G extracellular-domain construct (Outside), and Flag-tagged CVS11-G intracellular-domain construct (Inside) groups. Flag signals are shown in green, Map2-positive neurons in red, and nuclei counterstained with DAPI in blue. Scale bars, 50 μm; (**b**) Relative Expression Levels of *Vamp2* mRNA measured by RT-qPCR. Relative mRNA abundance is calculated using the $2^{-\Delta\Delta Ct}$ method. Data are presented as mean ± SD. Statistical comparisons are performed by one-way ANOVA followed by a post hoc test; (c) Relative cell viability 48 hours after transfection, as determined by the CCK-8 assay (normalized to 100% for the Flag empty vector control). Data are presented as mean ± SD from three biological replicates, and each biological replicate in the RT-qPCR experiment is measured in three technical replicates. One-way ANOVA followed by post hoc tests was used. $p < 0.05$ was considered statistically significant for all comparisons. Symbols indicate significance levels: *$p < 0.05$.

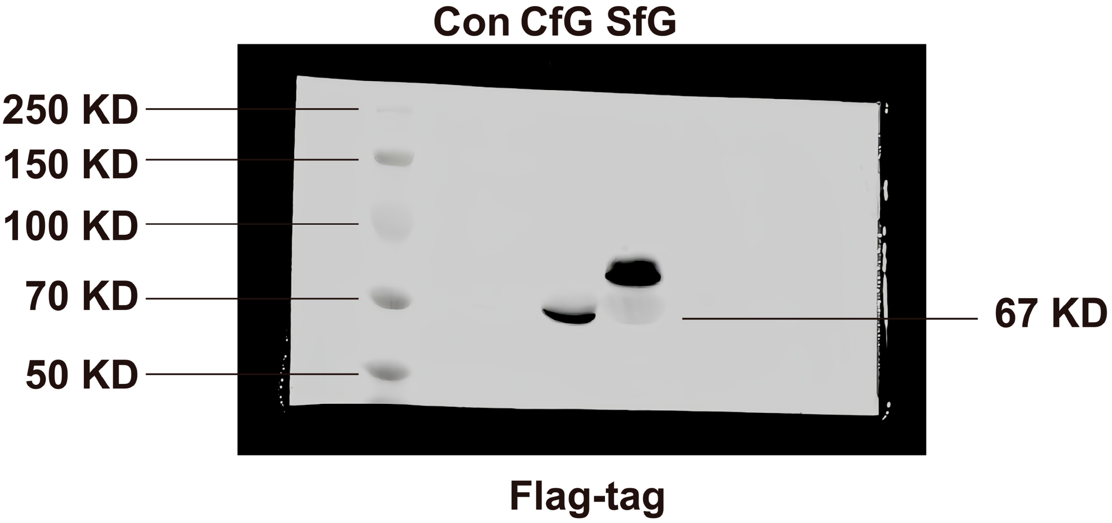

Figure S4. Western blot detection of rLBNSE-SfG and rLBNSE-CfG using anti-Flag antibodies. Lane 1: marker; Lane 2: Control; Lane 3: rLBNSE-CfG; Lane 4: rLBNSE-SfG.

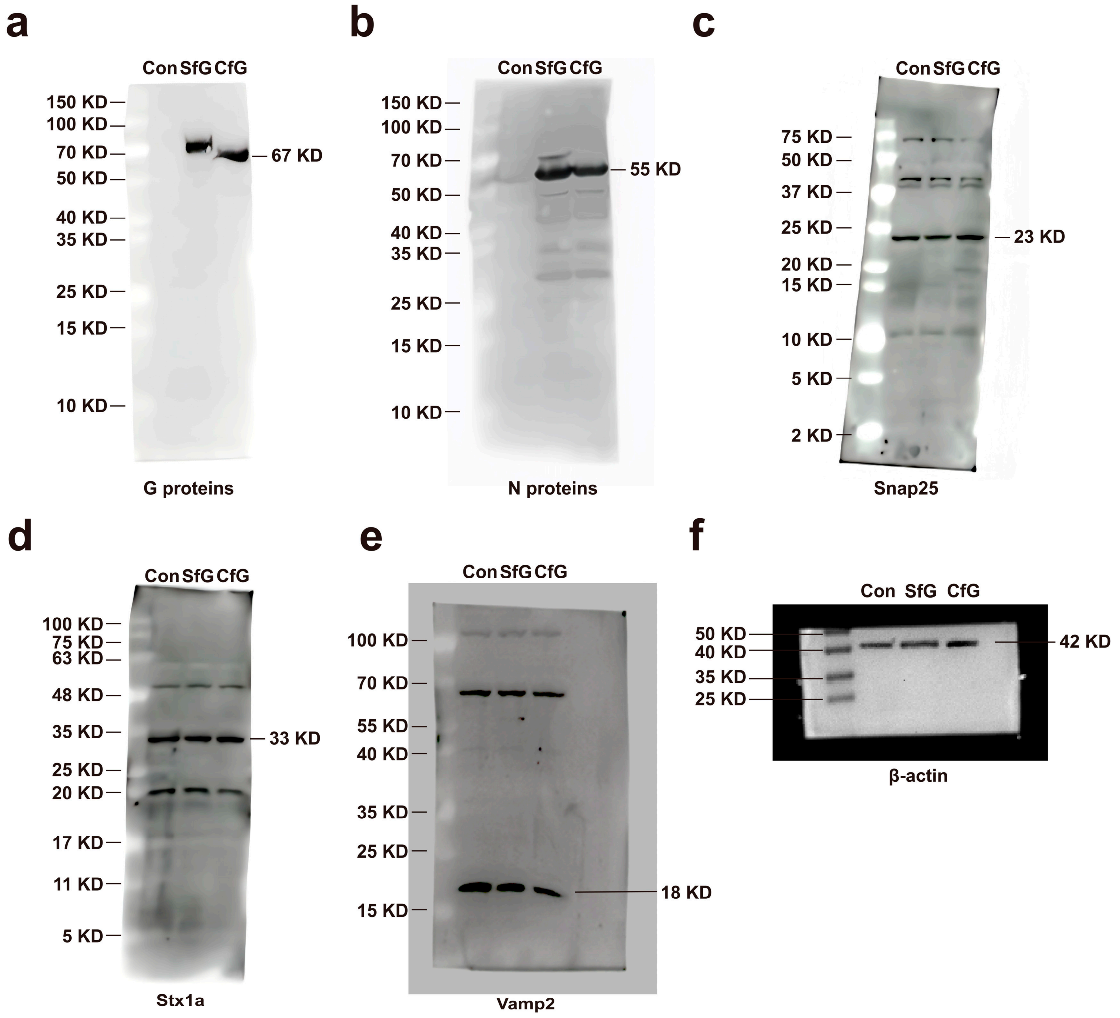

Figure S5. Western blot of G protein, N protein, Snap25, Stx1a, and Vamp2 in Control, rLBNSE-SfG, and rLBNSE-CfG groups. (**a**) G protein; (**b**) N protein; (**c**) Snap25; (**d**) Stx1a; (**e**) Vamp2; (**f**) β-actin. Lane 1: marker; Lane 2: Control; Lane 3: rLBNSE-SfG; Lane 4: rLBNSE-CfG.

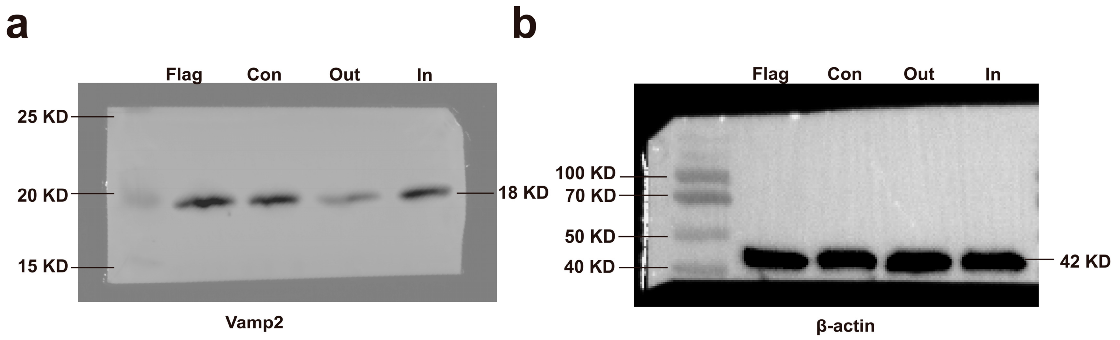

Figure S6. CVS11 G Protein Extracellular Domain Regulates Vamp2 Expression in Primary Mouse Neurons. (**a**) Vamp2; (**b**) β-actin. Lane 1: marker; Lane 2: pCDNA3.1-Flag; Lane 3: Control; Lane 4: pCDNA3.1-CVS11-G-outside; Lane 5: pCDNA3.1-CVS11-G-inside.
